# The Helicase Aquarius/EMB-4 Is Required to Overcome Intronic Barriers to Allow Nuclear RNAi Pathways to Heritably Silence Transcription

**DOI:** 10.1016/j.devcel.2017.07.002

**Published:** 2017-08-07

**Authors:** Alper Akay, Tomas Di Domenico, Kin M. Suen, Amena Nabih, Guillermo E. Parada, Mark Larance, Ragini Medhi, Ahmet C. Berkyurek, Xinlian Zhang, Christopher J. Wedeles, Konrad L.M. Rudolph, Jan Engelhardt, Martin Hemberg, Ping Ma, Angus I. Lamond, Julie M. Claycomb, Eric A. Miska

**Affiliations:** 1 Wellcome Trust Cancer Research UK Gurdon Institute, University of Cambridge, Tennis Court Road, Cambridge CB2 1QN, UK; 2 Department of Genetics, University of Cambridge, Downing Street, Cambridge CB2 3EH, UK; 3 Wellcome Trust Sanger Institute, Wellcome Trust Genome Campus, Cambridge CB10 1SA, UK; 4 Department of Molecular Genetics, University of Toronto, Toronto, ON M5S 1A8, Canada; 5 Centre for Gene Regulation and Expression, School of Life Sciences, University of Dundee, Dundee DD1 5EH, UK; 6 Department of Statistics, University of Georgia, Athens, GA 30602, USA; 7 Bioinformatics Group, Department of Computer Science, Interdisciplinary Center for Bioinformatics, University of Leipzig, Haertelstraße 16-18, Leipzig 04107, Germany

**Keywords:** transposable elements, RNAi, epigenetic inheritance, piRNA, Piwi, nuclear RNAi, RNA processing, transcription, splicing, *C. elegans*

## Abstract

Small RNAs play a crucial role in genome defense against transposable elements and guide Argonaute proteins to nascent RNA transcripts to induce co-transcriptional gene silencing. However, the molecular basis of this process remains unknown. Here, we identify the conserved RNA helicase Aquarius/EMB-4 as a direct and essential link between small RNA pathways and the transcriptional machinery in *Caenorhabditis elegans*. Aquarius physically interacts with the germline Argonaute HRDE-1. Aquarius is required to initiate small-RNA-induced heritable gene silencing. HRDE-1 and Aquarius silence overlapping sets of genes and transposable elements. Surprisingly, removal of introns from a target gene abolishes the requirement for Aquarius, but not HRDE-1, for small RNA-dependent gene silencing. We conclude that Aquarius allows small RNA pathways to compete for access to nascent transcripts undergoing co-transcriptional splicing in order to detect and silence transposable elements. Thus, Aquarius and HRDE-1 act as gatekeepers coordinating gene expression and genome defense.

## Highlights

• Nuclear Argonaute HRDE-1 physically interacts with the helicase EMB-4/AQR
• EMB-4/AQR is required for heritable silencing of genes and transposable elements
• Intronic sequences form a barrier to nuclear RNAi pathway
• EMB-4/AQR removes intronic barriers for efficient silencing by HRDE-1

## Graphical Abstract

**Figure:**
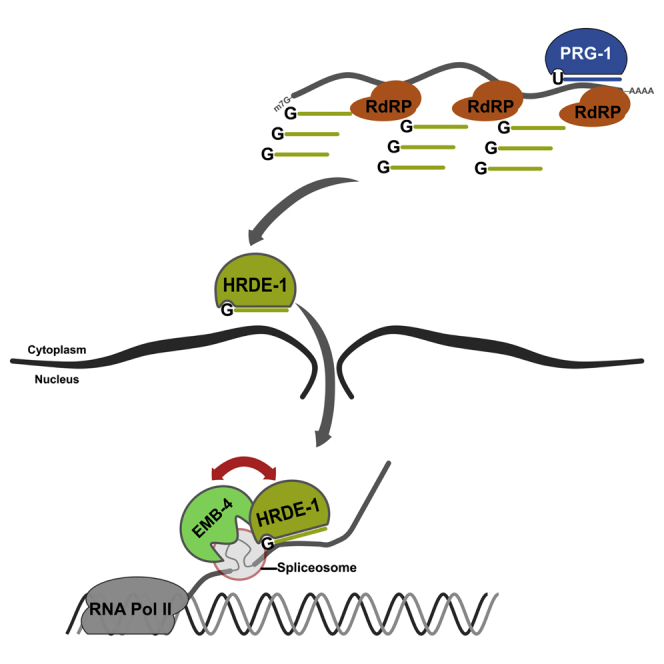

## Teaser

Nuclear RNAi is essential for heritable silencing in many organisms. Akay et al. show that RNA helicase EMB-4/AQR physically interacts with nuclear Argonaute HRDE-1 and bridges the small RNA pathway to transcriptional silencing. They further show that introns can form a barrier to transcriptional silencing, which EMB-4/AQR can overcome.

## Introduction

Transposable elements (TEs) are a feature of almost all eukaryotic genomes, which if left unchecked present a considerable danger to genome integrity and species survival. Therefore, organisms have evolved robust pathways to silence the expression of transposable elements and to restrict their mobility (Slotkin and Martienssen, 2007). Among these, DNA and chromatin modification and small RNA (sRNA)-mediated silencing are ancient and conserved mechanisms. sRNA pathways in eukaryotes are all related to RNAi mechanisms (Fire et al., 1998): 21–32-nt sRNAs are bound by Argonaute superfamily proteins, interact with target RNAs through Watson-Crick base pairing, and initiate silencing of these targets. Such sRNA-mediated silencing can be post-transcriptional on mRNAs in the cytoplasm (PTGS) or co-transcriptional on nascent transcripts in the nucleus (coTGS). The latter provides the potential to couple sRNA-mediated silencing to DNA and chromatin-based gene regulatory pathways. The mechanisms by which such sRNA-mediated silencing affects DNA methylation and/or chromatin modification remain largely unknown, particularly in animals (Holoch and Moazed, 2015, Weick and Miska, 2014).

Most organisms have also evolved sRNA amplification systems to enhance sRNA-mediated silencing. The ancestral amplification mechanism is based on RNA-dependent RNA polymerases (RdRPs), which use target RNAs as templates to generate secondary sRNAs. Some animals, including mammals, have lost RdRPs but have instead evolved the Ping-Pong amplification system (Brennecke et al., 2007, Gunawardane et al., 2007). The Ping-Pong pathway amplifies Piwi-interacting RNAs (piRNAs), an animal-specific class of sRNAs, through double-stranded target RNA intermediates and Argonaute RNase activity (RNA slicing). piRNAs are named after a subfamily of Argonautes, the Piwi proteins (Cox et al., 1998). Piwi proteins and piRNAs act in TE silencing in the germline of many animals. Another distinction of piRNAs is that they act in *trans*, whereby piRNAs generated from genomic clusters silence TEs throughout the genome. piRNA clusters can be thought of as a memory of TEs within the genome of an organism analogous to the guide RNA cluster/CRISPR systems of prokaryotes (Wiedenheft et al., 2012). Like other sRNAs, piRNAs have been shown to act through PTGS and coTGS mechanisms in nematodes, insects, fish, and mice (Holoch and Moazed, 2015, Weick and Miska, 2014).

*Caenorhabditis elegans* has both PTGS and coTGS mechanisms in the soma and the germline (Weick and Miska, 2014). In the germline, sRNAs and piRNAs can initiate coTGS through a two-step mechanism. *C. elegans* piRNAs are 21-nt RNAs with a 5′ uracil (21U-RNAs) that are bound by the PRG-1 Piwi protein in the germline cytoplasm (Batista et al., 2008, Das et al., 2008, Wang and Reinke, 2008). Once a PRG-1/piRNA complex has recognized a target RNA it recruits an RdRP-containing complex to generate 22-nt antisense sRNAs with a 5′ guanine (22G-RNAs) (Bagijn et al., 2012, Pak and Fire, 2007). 22G-RNAs are then bound by the Argonaute HRDE-1 and imported into the nucleus (Ashe et al., 2012, Buckley et al., 2012). An HRDE-1/22G-RNA complex is thought to directly interact with nascent transcripts. Genetic screens have identified several additional factors that are required for HRDE-1-mediated coTGS including NRDE factors (NRDE-1/-2/-4), the H3K9 histone methyltransferases SET-25 and SET-32, and the heterochromatin protein 1 (HP1) homolog HPL-2 (Ashe et al., 2012, Buckley et al., 2012, Burkhart et al., 2011, Guang et al., 2008, Shirayama et al., 2012). However, how HRDE-1 links sRNA-mediated silencing to coTGS and chromatin modifications remains unknown.

Transcription and mobility of TEs is actively suppressed by this two-step piRNA/22G-RNA coTGS system in *C. elegans*. Interestingly, piRNA- and sRNA-mediated coTGS can last for multiple generations (Ashe et al., 2012, Luteijn et al., 2012, Shirayama et al., 2012) and can be the source of non-genetic transgenerational effects (Ashe et al., 2012, Jobson et al., 2015, Ni et al., 2016, Rechavi et al., 2014), and loss of genes in the piRNA and the 22G-RNA pathway result in a multi-generational loss of fertility (de Albuquerque et al., 2015, Buckley et al., 2012, Phillips et al., 2015, Simon et al., 2014).

Eukaryotic mRNA transcription is a complex, multi-step process. First, the RNA polymerase II holoenzyme assembles on genomic DNA at a transcription start site. Second, nascent transcripts are produced by the elongating RNA polymerase II. Third, nascent RNA transcripts are processed to mature mRNAs through the assembly of multiple large RNA protein (RNP) complexes to carry out 5′ capping (Gonatopoulos-Pournatzis and Cowling, 2014), splicing (Carrillo Oesterreich et al., 2016), and 3′ poly(A) tailing (Shatkin and Manley, 2000). All of these steps are required to protect transcripts from degradation and to ensure that mRNAs are successfully exported into the cytoplasm for protein translation. Of these co-transcriptional events splicing is probably the most complex of all, requiring more than 200 proteins and many non-coding RNAs (Nilsen, 2003, Wahl and Lührmann, 2015). Pre-mRNA splicing by the spliceosome is immediately followed by the assembly of a set of proteins known as the exon-junction complex (EJC). EJC functions in the export, localization, and translation of mRNAs. Assembly of different EJC components can determine the fate of mRNAs, and EJC can be considered a regulatory hub between transcription and translation (Le Hir et al., 2016).

Interestingly, most TEs are adapted to the host transcription and RNA-processing machineries, and exploit them for their own expression and mobility. Furthermore, TEs have been domesticated in such a way that their regulation can contribute to gene regulation (Cowley and Oakey, 2013, Rebollo et al., 2012).

Here, we used a proteomics approach to identify protein interactors of the germline Argonaute HRDE-1 in *C. elegans*. One HRDE-1 interacting factor is the conserved RNA helicase Aquarius/EMB-4. HRDE-1 and Aquarius/EMB-4 act to silence an overlapping set of TEs and TE-containing genes. Surprisingly, removal of introns from a coTGS target removes the requirement for Aquarius/EMB-4 in sRNA-mediated silencing. Thus, Aquarius/EMB-4 activity allows HRDE-1 access to the nascent RNA transcripts.

## Results

### SILAC Proteomics Identifies Protein Interactors of the Germline Nuclear Argonaute HRDE-1

**Figure 1:**
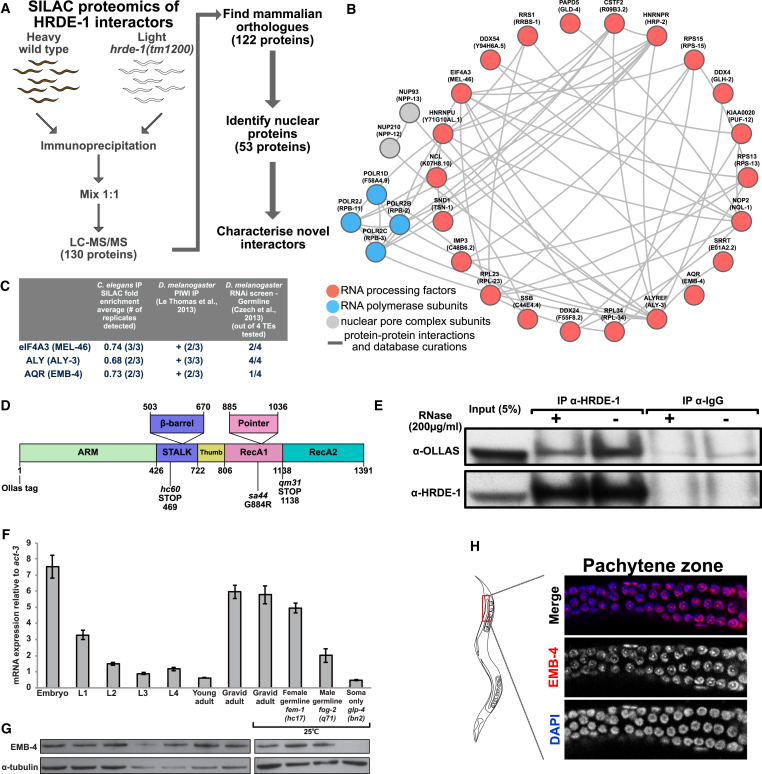
SILAC Proteomics Identifies Aquarius/EMB-4 as an Interactor of the Nuclear Argonaute HRDE-1 (A) SILAC labeling and IP scheme for wild-type (heavy labeled) and *hrde-1*(*tm1200*) mutants (light labeled) using anti-HRDE-1 antibodies. (B) Known protein interactions identified by the STRING database for mammalian orthologs of nuclear HRDE-1 interactors. Gray lines indicate known protein-protein interactions, red circles highlight known RNA-processing factors, blue circles highlight RNA pol II subunits, and gray circles highlight nuclear pore complex subunits. (C) Aquarius and two exon-junction complex proteins eIF4A3 and ALY are detected in HRDE-1 IPs (second column, mean log_2_ fold enrichment heavy/light and number of replicates detected) and in *D. melanogaster* PIWI IPs (third column, number of IPs detected/total number of IPs). The effect on TE desilencing in *D. melanogaster* upon RNAi knockdown (fourth column, number of TEs desilenced out of four tested). (D) EMB-4 domain structure based on the mammalian homolog Aquarius and the position of *emb-4* mutations used in this study. (E) Validation of protein-protein interactions between HRDE-1 and EMB-4 using anti-HRDE-1 antibodies to immunoprecipitate HRDE-1 complexes in CRISPR-tagged OLLAS-EMB-4 strain with or without RNase treatment (immunoglobulin G [IgG] negative control). (F) qRT-PCR analysis of *emb-4* mRNA expression at different developmental stages (embryo to gravid adult), in animals lacking sperm (female germline *fem-1*(*hc17*)), in animals lacking oocytes (male germline *fog-2*(*q71*)), and in animals lacking a germline (soma only *glp-4*(*bn2*)). *fem-1*(*hc17*), *fog-2*(*q71*), and *glp-4*(*bn2*) are temperature-sensitive mutants and were grown at 25°C alongside the wild-type gravid adult control. Error bars represent SD. (G) Western blot analysis of EMB-4 protein levels using anti-EMB-4 antibodies for the same conditions as in Figure 2A. (H) Localization of EMB-4 protein in the germline of adult animals. See also Figures S1 and S2.

To further our understanding of coTGS in animals and as a complement to genetic approaches, we sought to identify proteins interacting with the nuclear Argonaute HRDE-1 by protein immunoprecipitation (IP) coupled with SILAC (stable isotope labeling of amino acids in cell culture) proteomics (Ong et al., 2002), using the SILAC labeling for nematodes approach (Akay et al., 2013, Fredens et al., 2011, Larance et al., 2011) (Figure 1A). We produced protein extracts from wild-type young adult *C. elegans* grown on “heavy” isotopes and *hrde-1*(*tm1200*) mutant animals grown on “light” isotopes. We chose the young adult stage, as the germline is then fully developed and endogenous HRDE-1 expression is at its peak (Ashe et al., 2012). We then immunoprecipitated a 1:1 mix of protein extracts of both genotypes using an anti-HRDE-1 antibody followed by liquid chromatography-tandem mass spectrometry (LC/MS-MS). Based on three biological replicates, LC/MS-MS identified 130 candidate HRDE-1 interacting proteins. For further analysis and to take advantage of prior proteomics data from mammals, we focused on 53 proteins with human orthologs that are also classified as being nuclear (Uhlén et al., 2015, UniProt Consortium, 2015). We used the STRING database (Szklarczyk et al., 2015) to identify known protein complexes between these factors. Twenty-eight of these 53 proteins were connected with each other based on known interactions, and the majority of the proteins represent RNA processing factors, RNA polymerase II subunits, and nuclear pore complex components (Figures 1B and S1A). In the fruit fly *Drosophila melanogaster*, the Piwi protein has a function analogous to that of HRDE-1 in coTGS in the germline (Weick and Miska, 2014). Interestingly, eight proteins identified in our HRDE-1 IPs were also found in *D. melanogaster* PIWI IPs. In addition, 13 of our HRDE-1 interactors were hits in genome-wide RNAi screens for regulators of transposable elements (Figure 1C and Table S1) (Czech et al., 2013, Le Thomas et al., 2013). However, the role of these interactors in the sRNA pathway is currently not understood in any organism. Overall, our SILAC-based HRDE-1 proteomics identified a set of conserved proteins that might bridge sRNA pathways, RNA processing, and TE silencing.

### The Aquarius RNA Helicase Ortholog EMB-4 Is Nuclear and Germline Enriched and Interacts with HRDE-1

Of the candidate HRDE-1 protein interactors, we were particularly curious about EMB-4 as its interaction with nuclear Argonaute proteins that appeared to be conserved in *D. melanogaster* (Figure 1C). EMB-4 is the *C. elegans* ortholog of Aquarius, or AQR, a large scaffolding protein that includes an Armadillo repeat and two RecA helicase domains (Figure 1D). Aquarius/EMB-4 is conserved in all eukaryotes with an intact RNAi pathway, including the fission yeast *Schizosaccharomyces pombe*, but not *Saccharomyces cerevisiae*, which lacks RNAi (Drinnenberg et al., 2011) (Figures S1A–S1C). As Aquarius has a conserved role in nascent RNA processing in eukaryotes (Figure S1D) (De et al., 2015, Wahl and Lührmann, 2015), we wondered whether Aquarius/EMB-4 might provide new insights into the interface between the nuclear RNAi pathway and the general RNA-processing machinery. Therefore, we validated the interaction between HRDE-1 and EMB-4 independently. First, we generated an N-terminal epitope-tagged version of EMB-4, *in vivo*, using CRISPR/Cas9 genome engineering (Paix et al., 2015, Wiedenheft et al., 2012) (Ollas tag, Figure 1D). We then tested the interaction between HRDE-1 and EMB-4-Ollas using an anti-Ollas antibody (Park et al., 2008) and an anti-HRDE-1 antibody (Ashe et al., 2012), through IP followed by western blotting. As shown in Figure 1E, we found that EMB-4-Ollas interacts specifically with HRDE-1 in whole-cell extracts (IP α-HRDE-1(−) RNase treatment, Figures S1E and S1F). A significant portion of the EMB-4/HRDE-1 interaction remains intact upon strong RNase treatment (Figure 1E, IP α-HRDE-1(+) RNase treatment, Figure S1F), consistent with a model in which EMB-4 binds directly to HRDE-1 but with interaction being stabilized on the nascent RNA transcript. In addition, we validated this interaction using an anti-EMB-4 antibody and an FLAG-tagged version of HRDE-1 generated by MosSCI transgenesis (Frøkjær-Jensen et al., 2008, Shirayama et al., 2012) (Figures S1G and S1H). Next, we asked whether EMB-4 is co-expressed with HRDE-1 *in vivo*. We found that *emb-4* mRNA was highly enriched in the germline, embryo, and early larval stages of *C. elegans* (Figure 1F). Using an anti-EMB-4 antibody, we observed the same for the endogenous EMB-4 protein (Figure 1G). Taking advantage of temperature-sensitive germline mutants of *C. elegans*, we found that in gravid adult animals the majority of EMB-4 mRNA and protein is germline restricted (Figures 1F and 1G). This is of particular interest, as HRDE-1 expression is germline specific (Ashe et al., 2012). Within the germline, we find that EMB-4 is highly enriched in germline nuclei and closely associated with chromatin in the mitotic, transition, and pachytene region of the germline as well as in oocytes (Figures 1H, S2A, and S2B). Mutations in *hrde-1* do not affect the nuclear localization of EMB-4, in line with the general role of EMB-4 in pre-mRNA processing. Similarly, mutations in *emb-4* do not change the overall nuclear localization of HRDE-1 (Figure S2B). Our observations are in agreement with the findings of the related work in this issue of *Developmental Cell* by Tyc et al. (2017). We conclude that EMB-4 is expressed in the germline nuclei and is a bona fide HRDE-1 interacting protein.

### Aquarius/EMB-4 Is Required for piRNA-Mediated Co-transcriptional Gene Silencing

**Figure 2:**
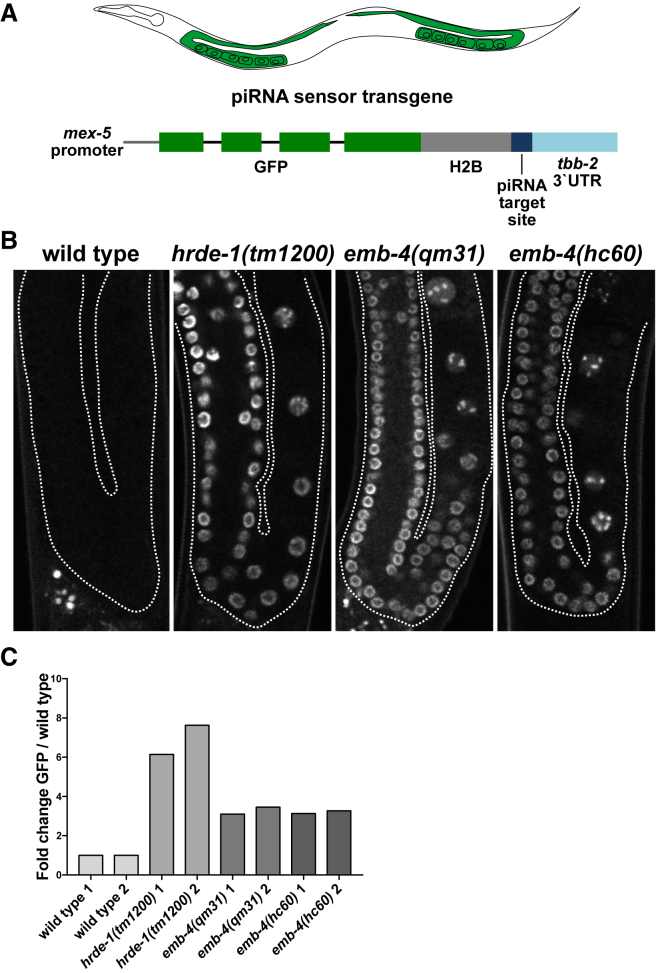
Aquarius/EMB-4 Is Required for the piRNA-Mediated Silencing of a Sensor Transgene (A) piRNA sensor transgene and its expression pattern in *C. elegans* germline. (B) Fluorescent microscope images of wild-type and mutant animal germlines with an integrated single-copy piRNA sensor transgene (germline boundaries are marked by white dotted lines). (C) qRT-PCR analysis of GFP expression in animals described in Figure 3B (two biological replicates with at least two technical replicates). See also Figure S3.

We examined whether, like the Argonaute HRDE-1, Aquarius/EMB-4 is required for coTGS in the germline. For this purpose we used a piRNA sensor strain we generated previously (Bagijn et al., 2012). The piRNA sensor (*mjIs144*) is a transgene that drives the expression of a GFP histone H2B fusion protein through the germline-specific *mex-5* gene promoter and is inserted as a single copy on chromosome II. In addition, the piRNA sensor contains a 21-nt sequence perfectly complementary to the endogenous piRNA 21UR-1 (Figure 2A). In otherwise wild-type animals, the piRNA sensor is silenced by piRNA-mediated coTGS. However, mutations in the piRNA or the germline nuclear RNAi pathway, e.g., *prg-1* (Bagijn et al., 2012), *prde-1* (Weick et al., 2014), or *hrde-1* (Ashe et al., 2012), lead to reactivation of the piRNA sensor. Taking advantage of two previously isolated alleles of *emb-4* (Checchi and Kelly, 2006, Katic and Greenwald, 2006), we tested the requirement of EMB-4 in piRNA-mediated gene silencing. We observed that two null mutants of *emb-4* desilenced the piRNA sensor in the germline of *C. elegans* as indicated by GFP expression in germline nuclei, as did *hrde-1* as the positive control, while the piRNA sensor was silenced in otherwise wild-type animals (Figures 2B and S3A). We also quantified mRNA levels of piRNA sensor expression in these mutant backgrounds, which confirmed the desilencing in *emb-4* and *hrde-1* mutant backgrounds (Figure 2C). Finally, we carried out an analogous experiment using an independent transgene targeted by the piRNA pathway (*ccSi1504*, gift from C. Frøkjær-Jensen). *ccSi1504* is integrated on chromosome V and expressed under *smu-1* promoter, utilizes *smu-1* introns, and contains SV40 and EGL-13 nuclear localization signals. Both *emb-4* and *hrde-1* mutants desilenced the *ccSi1504* transgene similarly to the *mjIs144* transgene (Figures S3B and S3C). Our results demonstrate that EMB-4 is an essential factor for piRNA-mediated coTGS in the germline.

### A Functional RNA Helicase Domain of Aquarius/EMB-4 Is Required to Establish Co-transcriptional Gene Silencing

**Figure 3:**
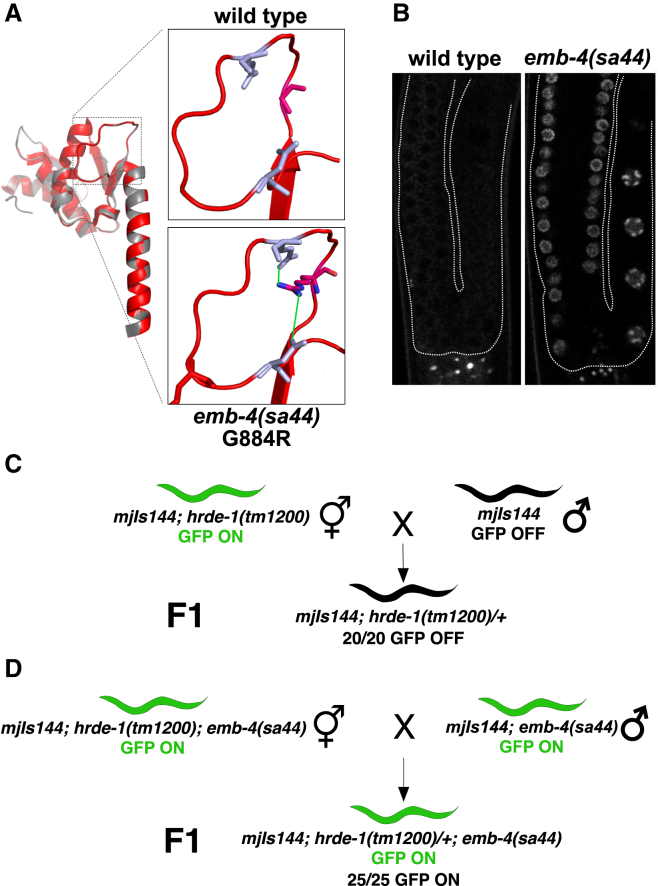
RNA Helicase Domain of Aquarius/EMB-4 Is Required for the Establishment of Transcriptional Gene Silencing (A) Structural alignment of the RecA1 and the pointer domains of human Aquarius (white) with *C. elegans* EMB-4 (red). Blown-up region shows the location of the G884R substitution found in *emb-4*(*sa44*) strain. G884R affects the loop region of the helicase domain. Green lines show possible salt bridge interactions between the amino acids. (B) Fluorescent images of wild-type and *emb-4*(*sa44*) germlines with the integrated piRNA sensor transgene (germline boundaries are marked by white dotted lines). (C and D) Scheme of genetic crosses showing the effect of *emb-4*(*sa44*) mutation during the establishment of gene silencing. (C) In the control cross, a wild-type copy of *hrde-1*(+) in the F1 heterozygous animals re-establishes complete piRNA sensor silencing. (D) Wild-type copy of *hrde-1* fails to establish piRNA sensor silencing in the *emb-4*(*sa44*) homozygous background (number of assayed F1 progeny is indicated below each cross). See also Figure S4.

The molecular function of Aquarius/EMB-4 *in vivo* remains unknown in any organism. However, previous studies identified Aquarius as intron binding and associated with spliceosome and EJC recruitment (Hirose et al., 2006, Ideue et al., 2007). *In vitro* experiments suggested a role for the ATPase activity of the RecA1 helicase domain of human Aquarius/AQR in RNA unwinding and spliceosomal complex assembly (De et al., 2015). We noted that the previously isolated allele *sa44* of Aquarius/EMB-4 in *C. elegans* is a missense mutation that induces a G884R substitution in the RecA1 helicase domain (Figure 3A) (Katic and Greenwald, 2006). *emb-4*(*sa44*) was identified in a genetic suppressor screen and lacks the embryonic lethality associated with null mutants of *emb-4* (Checchi and Kelly, 2006, Katic and Greenwald, 2006). Based on the structural alignment between human Aquarius/AQR, UPF1, and *C. elegans* EMB-4 (Figures S4A–S4C), G884R likely affects RNA binding and, thus, helicase activity (Figures 3A and S4D), without affecting the overall protein levels of Aquarius/EMB-4 (Figure S4E) (Chakrabarti et al., 2011, Cheng et al., 2007, De et al., 2015). Interestingly, *emb-4*(*sa44*) animals desilence the piRNA sensor similarly to *emb-4*(*null*) mutations (Figure 3B). Thus, Aquarius/EMB-4 helicase activity is required for co-transcriptional gene silencing. We next asked whether Aquarius/EMB-4 is required for the establishment and/or the maintenance of co-transcriptional gene silencing. Taking advantage of the *emb-4*(*sa44*) mutants, we addressed this using genetic crosses (Figures 3C and 3D). When *hrde-1*(*tm1200*);*mjIs144* (piRNA sensor) animals with a desilenced piRNA sensor are crossed to *mjIs144* animals generating F1 animals heterozygous for *hrde-1*, piRNA sensor silencing is restored (Figure 3C). However, when carrying out an analogous cross of *hrde-1*(*tm1200*);*mjIs144*;*emb-4*(*sa44*) and *mjIs144*;*emb-4*(*sa44*) animals, piRNA sensor silencing is not restored (Figure 3D). We conclude that Aquarius/EMB-4 is required for the establishment of co-transcriptional gene silencing.

### Aquarius/EMB-4 Is Required for sRNA-Dependent Co-transcriptional Silencing of Endogenous Genes

**Figure 4:**
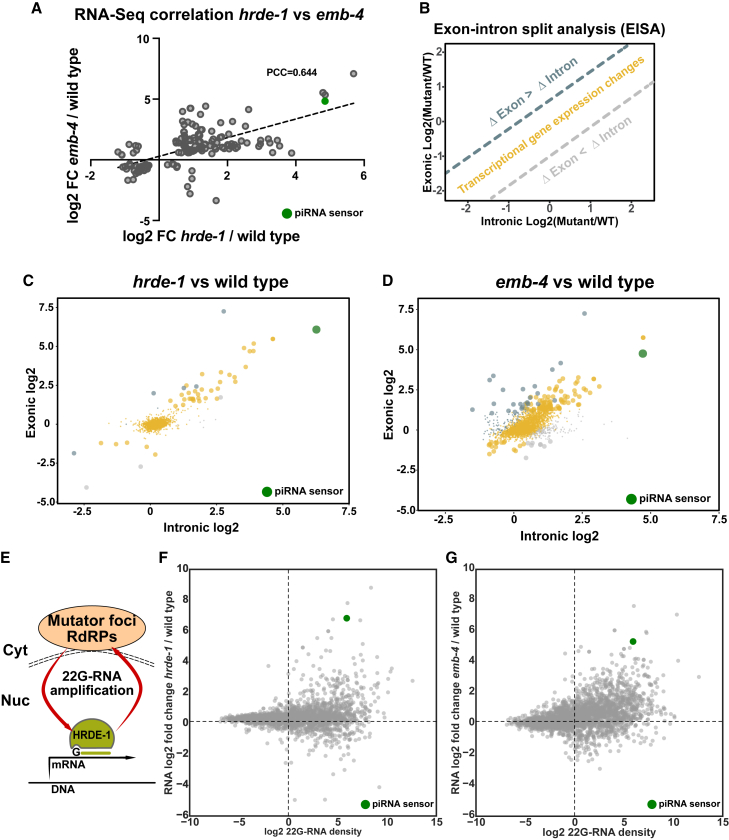
EMB-4 and HRDE-1 Are Required for Transcriptional Silencing of Genes and Transposable Elements (A) Log_2_ fold change values of genes, which show significant expression change (adjusted p ≤ 0.05) in both *hrde-1* and *emb-4* mutants (151 genes in total), are plotted against each mutant background (dashed line, linear fit curve; PCC, Pearson's correlation coefficient; green dot, piRNA sensor transgene). (B) Exon-intron split analysis (EISA) for comparison of transcriptional and post-transcriptional gene expression changes. (C and D) Transcriptional and post-transcriptional gene expression changes in *hrde-1* (C) and *emb-4* (D) are colored as in (B). Significant gene expression changes are highlighted by larger dot size (large dots denote mRNA log_2_ fold change ≥ 1, p ≤ 0.05). piRNA sensor transgene is highlighted by the green dot. (E) Model showing 22G-RNA amplification in mutator foci, which requires HRDE-1 for 22G-RNA transport and stability. (F and G) Comparison of RNA log_2_ fold change in *hrde-1* and *emb-4* mutants with log_2_ 22G-RNA density in wild-type animals (22G-RNA density = 22G-RNA count in HRDE-1 IP [wild-type]/RNA reads per kilobase per million mapped reads [wild-type]). piRNA sensor transgene is highlighted by the green dot. See also Figure S5.

Next, we aimed to understand the role of Aquarius/EMB-4 on the endogenous transcriptome. We generated total RNA and sRNA expression profiles using high-throughput sequencing of wild-type animals and *hrde-1* and *emb-4* mutant animals. In our RNA sequencing (RNA-seq) data, when only the genes that show significant expression change in both *hrde-1* and *emb-4* mutants are considered, the majority of these genes show upregulation as opposed to downregulation and there is a significant correlation in gene expression levels between *emb-4* and *hrde-1* mutants (Figure 4A and Table S2, PCC = 0.644, p < 0.0001). Next, we took advantage of the fact that total RNA-seq contains intronic sequence reads in addition to exonic sequence reads. Considering intronic reads as a measure of nascent RNA transcription, one can therefore infer changes of transcription rate in addition to steady-state mRNA levels. Such a method was recently reported as exon-intron split analysis (EISA) (Figure 4B) (Gaidatzis et al., 2015). Plotting the logarithm of the ratio of intronic reads from wild-type and mutant samples against the logarithm of the ratio of exonic reads of the same datasets, one can infer the type of gene expression change between the samples; that is, genes that vary in their transcription rate are aligned on the diagonal (yellow, Figures 4B–4D), positive post-transcriptional regulation is above the diagonal (blue, Figures 4B–4D), and negative post-transcriptional regulation is below the diagonal (gray, Figures 4B–4D). Comparing three biological replicates each of wild-type animals, *hrde-1*, and *emb-4* mutants (three replicates each of the *qm31* and *hc60* alleles, combined), we found that the majority of gene expression changes in *hrde-1* and *emb-4* mutants are due to changes in transcription rate (Figures 4C and 4D, percent transcriptional changes: 83% in *hrde-1* and 73% in *emb-4*; transcriptionally up/down: 34/5 in *hrde-1*, 100/16 in *emb-4*). Similarly, the piRNA sensor showed increased transcription rates in both *hrde-1* and *emb-4* mutants (Figures 4C and 4D, green dot). These findings are consistent with the known role of HRDE-1 in co-transcriptional gene silencing (Ashe et al., 2012, Buckley et al., 2012). We conclude that EMB-4 similarly affects transcription rate, which is consistent with a model of EMB-4 and HRDE-1 acting together in co-transcriptional gene silencing. In addition, we conclude that EMB-4 has no major impact on pre-mRNA splicing, as only a few genes showed a relative increase in intronic reads (Figure 4D, gray dots).

Furthermore, we considered sRNA expression alongside total RNA expression to focus on the direct targets of co-transcriptional gene silencing. As expected, *hrde-1* and *emb-4* mutants do not show any change in piRNA population (21U-RNAs), indicating that the upstream piRNA pathway remains intact in these mutant backgrounds (Figure S5A). HRDE-1-bound sRNAs are 22G-RNAs, antisense to their target RNAs and generated by RdRPs at perinuclear foci called mutator bodies (Figure 4E). HRDE-1 is required for the stability and amplification of 22G-RNAs in the germline (Ashe et al., 2012, Buckley et al., 2012, Sapetschnig et al., 2015). We therefore calculated the 22G-RNA density for genes with a matching 22G-RNA in HRDE-1 IPs, as the ratio of HRDE-1-bound 22G-RNAs in the wild-type and the expression levels of their target RNAs (Gerson-Gurwitz et al., 2016, Sapetschnig et al., 2015). We found that genes with high 22G-RNA density in wild-type animals tended to be upregulated in both *hrde-1* and *emb-4* mutants (Figures 4F and 4G).

### Aquarius/EMB-4 Is Required for Silencing of Transposable Elements

**Figure 5:**
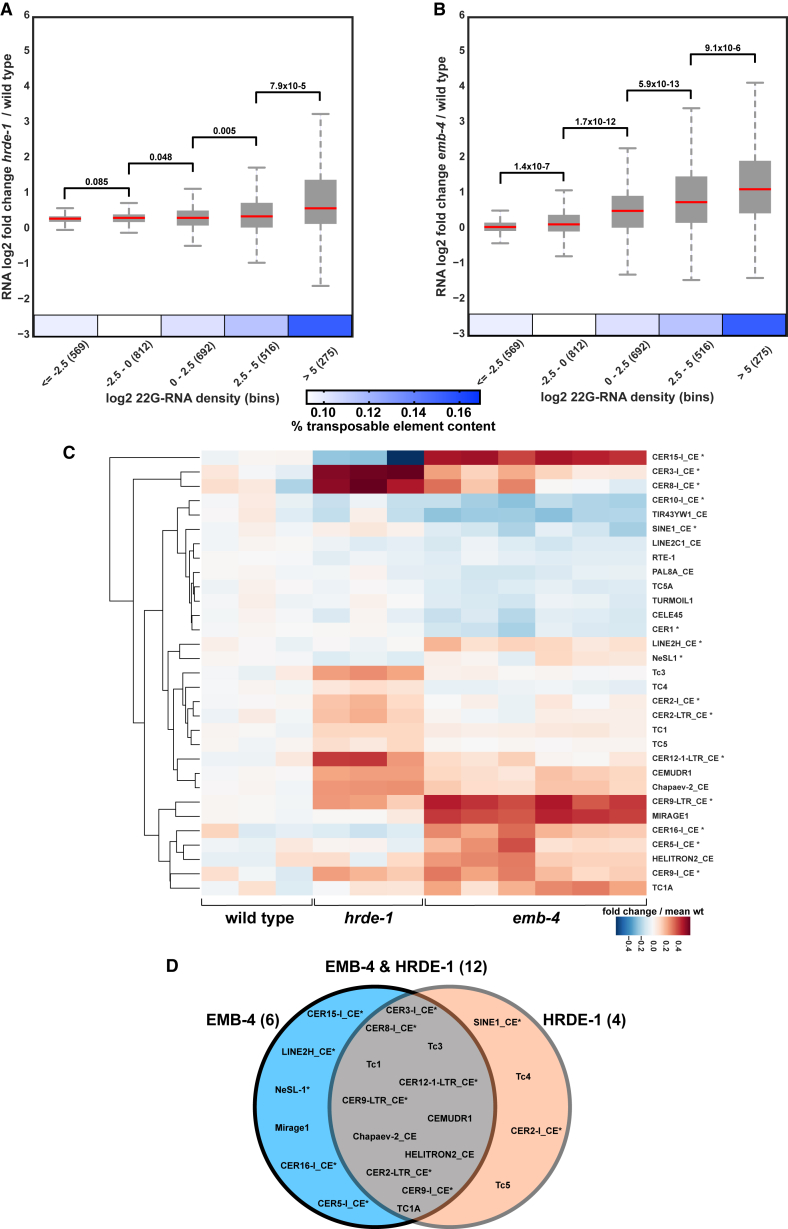
HRDE-1 and EMB-4 Are Required for the Suppression of Multiple Transposable Element Families (A and B) Genes in Figures 4F and 4G are grouped into bins of increasing 22G-RNA density as shown on the x axis (number of genes in each bin is shown in parentheses, boxes show the lower and upper quartiles, red line shows the median, and p values of two-sample t test are shown above the box plots). Heatmap shows percent abundance of transposable elements in each 22G-RNA bin. (C) Heatmap showing RNA fold change of transposable element families in wild-type, *hrde-1*, and *emb-4* mutants compared with mean wild-type levels. Asterisks indicate retro-element families. (D) Venn diagram summarizing the overlap of upregulated transposable element families in *hrde-1* and *emb-4* mutant animals as shown in (C).

For further analysis we grouped all genes from Figures 4F and 4G into bins of increasing 22G-RNA density (Figures 5A and 5B, bins 1–5, 275–569 genes per bin) and found a number of correlating features. HRDE-1-bound 22G-RNAs are enriched in germline 22G-RNA targets and depleted from somatic 22G-RNA targets, as expected (Figure S5B) (Gu et al., 2009). In addition, 22G-RNA density correlates positively with piRNA targets and targets of the WAGO-1 pathway, which is known to overlap with HRDE-1 targets (Figure S5B) (Lee et al., 2012). Finally, ERGO-1, ALG-3/4, and CSR-1 Argonautes are all required for additional endogenous RNAi pathways that also produce 22G-RNAs (Claycomb et al., 2009, Conine et al., 2010, Vasale et al., 2010). We found that HRDE-1-bound 22G-RNAs were overall depleted from ERGO-1 and ALG-3/4 targets, and the density of HRDE-1-bound 22G-RNAs inversely correlated with CSR-1 targets (Figure S5B).

**Figure 6:**
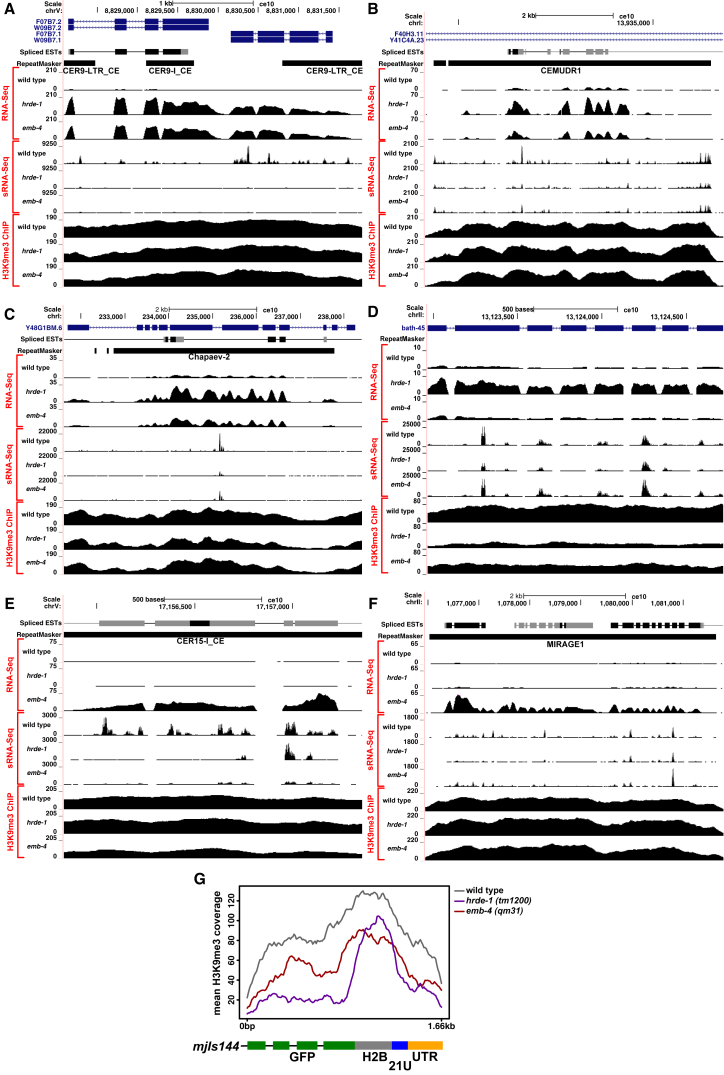
Exemplary Upregulated Regions with mRNA, 22G-RNA, and H3K9me3 Chromatin IP Profiles in *hrde-1* and *emb-4* Mutants (A–F) CER9-LTR_CE/CER9-I_CE retro-elements (A), CEMUDR1 transposable element (B), Chapaev-2 transposable element (C), piRNA target gene *bath-45* (D), CER15-I_CE retro-element (E), and Mirage1 transposable element (F). Repeatmasker tracks show transposable elements, and spliced EST tracks show evidence for canonical introns (UCSC genome browser). (G) H3K9me3 chromatin IP profile of piRNA sensor transgene.

HRDE-1-bound 22G-RNAs are known to target transposable elements (Ni et al., 2014). Indeed, genes with the highest 22G-RNA density also have the highest TE content and show the strongest mRNA upregulation both in *hrde-1* and *emb-4* mutant animals (Figures 5A and 5B). We therefore asked whether EMB-4, like HRDE-1, regulates transposable element expression. When grouping TEs into families, we found that several DNA transposons and retro-elements were overexpressed in *hrde-1* and/or *emb-4* mutants (Figure 5C). Out of 22 TE families that show upregulation, 12 are co-regulated by EMB-4 and HRDE-1, whereas 6 are upregulated specifically in *emb-4* mutants and 4 are upregulated specifically in *hrde-1* mutants (Figure 5D). For instance, CER9-I_CE, CER9-LTR_CE, CEMUDR1, and Chapaev-2 are strongly induced in both *hrde-1* and *emb-4* mutants. Furthermore, this RNA induction is accompanied by significant loss of 22G-RNAs to the same locus in both mutant backgrounds (Figures 6A–6C). On the other hand, *bath-45* is a piRNA pathway target gene with multiple piRNA target sites on all of its exons. *bath-45* mRNA levels were upregulated in *hrde-1* but not in *emb-4* mutant animals (Figure 6D). Similarly two TEs, CER15-I_CE and Mirage1, showed strong upregulation only in *emb-4* mutants (Figures 6E and 6F). Interestingly, CER15-I_CE is also upregulated in *hrde-1* mutants when the animals are subjected to heat stress (Ni et al., 2016). Altogether, we conclude that HRDE-1 and Aquarius/EMB-4 act together to silence many endogenous 22G-RNA target loci, genes, and transposable elements.

Chromatin-level silencing by histone modifications such as histone H3 lysine 9 trimethylation (H3K9me3) on the target loci has been proposed as a mechanism to maintain long-term silencing of sRNA targets in animals (Holoch and Moazed, 2015). In *C. elegans*, mutations in the histone methyltransferases *set-25* and *set-32* are sufficient to abolish the silencing of the piRNA sensor transgene (Ashe et al., 2012). We performed genome-wide H3K9me3 profiling by establishing the chipmentation method in *C. elegans* (Schmidl et al., 2015). Triplicate analysis of wild-type, *hrde-1*, and *emb-4* mutant animals show that H3K9me3 response is highly loci specific. For instance, H3K9me3 levels on the piRNA sensor, particularly on the GFP sequence, show strong reduction in *hrde-1* and *emb-4* mutant animals, which also correlate with the level of GFP mRNA upregulation in each mutant background (Figures 6G and 2C). Similarly, H3K9me3 levels are reduced on *bath-45*, *Chapaev-2*, and *CER15-I_CE* in mutant backgrounds that show mRNA upregulation in these loci (Figures 6C–6E). In contrast, H3K9me3 levels do not show much change on CER9-I_CE, CEMUDR1, or Mirage1 loci. These observations are in line with recent studies showing that methyltransferase mutants affect certain *C. elegans* sRNA pathways more than others (McMurchy et al., 2017, Zeller et al., 2016) and that the correlation between H3K9me3 levels and gene silencing can be highly gene and loci specific (Kalinava et al., 2017, Lev et al., 2017).

### Aquarius/EMB-4 Is Specifically Required to Silence Spliced Transcripts

**Figure 7:**
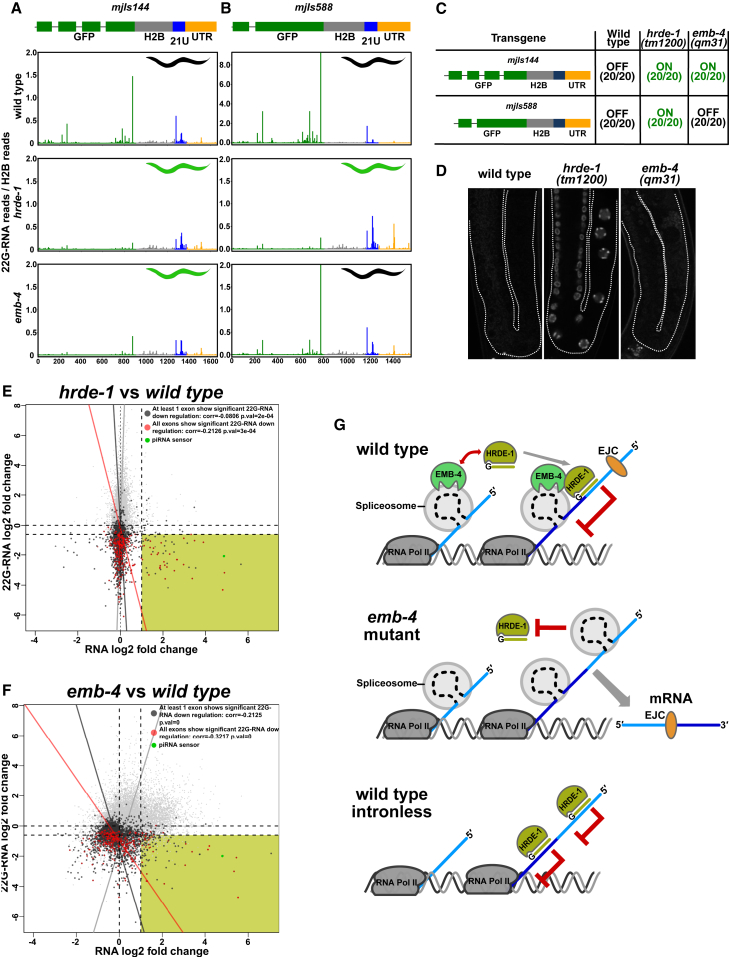
Aquarius/EMB-4 Acts to Remove Intronic Barriers to Transcriptional Gene Silencing (A and B) 22G-RNA profiles of the three-intron (A) and the single-intron (B) piRNA sensors in wild-type, *hrde-1*, and *emb-4* mutant animals (mean 22G-RNA abundance of three replicates) (y axis scale in wild-type animals of B is different from that of A; colors indicate different regions of the sensor transgene). (C) Silencing of the three-intron piRNA sensor (*mjIs144*) and the single-intron piRNA sensor (*mjIs588*) in wild-type and mutant animals (20 animals assayed for each condition). (D) Fluorescent microscope images of animals with the single-intron piRNA sensor (*mjIs588*). Germline boundaries are marked by the white dotted lines. (E and F) mRNA expression levels correlate negatively with 22G-RNA abundance and correlation increases when more exons are targeted by 22G-RNAs (gray line, all genes; black line, genes that show significant 22G-RNA change in one exon; red line, genes that show significant 22G-RNA change in all exons). The x axis shows log_2_ fold change of 22G-RNAs in mutants/wild-type and the y axis shows log_2_ fold change of mRNA in mutants/wild-type (correlation coefficient and p values are shown on graphs). (G) Model for Aquarius/EMB-4 function in intron-dependent transcriptional gene silencing.

As Aquarius is known to bind introns and is required for RNP remodeling during spliceosome and EJC assembly, we wondered whether Aquarius/EMB-4 is specifically required to allow co-transcriptional gene silencing on nascent transcripts undergoing splicing. To test this we took advantage of the piRNA sensor, which requires HRDE-1 and EMB-4 for co-transcriptional gene silencing (Figures 2, 3, and 4). We previously characterized 22G-RNA populations for the piRNA sensor in detail (Sapetschnig et al., 2015): upon initial piRNA targeting of the sensor transgene in the 3′ UTR, 22G-RNAs are generated proximal to the piRNA target site that are independent of HRDE-1 and the nuclear RNAi machinery and are not sufficient for piRNA silencing (Figure 7A, wild-type [blue bars]). Subsequently, HRDE-1-dependent 22G-RNAs spread along the whole length of the transcript, including the *GFP* coding region, to induce co-transcriptional gene silencing (Figure 7A, wild-type [green bars]). We find that loss of HRDE-1 or Aquarius/EMB-4 resulted in the loss of the majority of 22G-RNAs mapping specifically to the coding region of the piRNA sensor, consistent with HRDE-1 and Aquarius/EMB-4 acting together in nuclear RNAi (Figure 7A, *hrde-1* and *emb-4* [green bars]).

The piRNA sensor transgene (*mjIs144*) has three synthetic introns within the *GFP* gene sequence. These same introns are generally used by the community to promote efficient transgene expression in *C. elegans*. To test whether Aquarius/EMB-4 is specifically required for co-transcriptional gene silencing of transcripts undergoing splicing, we removed two of the three introns from the piRNA sensor transgene (*mjIs144*) to generate a new single-intron piRNA sensor integrated into the same chromosomal location (*mjIs588*) (Figure 7B). We retained the single remaining intron, as an intronless transgene is unlikely to be expressed in *C. elegans* (Fire et al., 1990). As expected, the single-intron piRNA sensor (*mjIs588*) was completely silenced in wild-type animals and fully desilenced in *hrde-1* mutant animals (Figures 7B–7D). In contrast, while the three-intron piRNA sensor required Aquarius/EMB-4 for silencing, the single-intron piRNA sensor did not (Figures 7B–7D). Importantly, GFP expression levels of the three-intron piRNA sensor and the single-intron piRNA sensor were similar in *hrde-1* mutants, confirming that the difference in Aquarius/EMB-4 dependence was not simply due to different transcription rates (Figure 7D). Comparing 22G-RNA populations for both piRNA sensors we found that the single-intron piRNA sensor accumulated 5- to 6-fold more 22G-RNAs compared with the three-intron piRNA sensor in wild-type animals (Figures 7A and 7B, wild-type panels: y axis range is different between graphs), indicating that introns can potentially form a barrier to nuclear RNAi by limiting 22G-RNA levels. On the other hand, most endogenous *C. elegans* genes have, on average, four introns (Michael and Manyuan, 1999), and intronic sequences are also present in transposable elements (Sijen and Plasterk, 2003) (Figure 6, spliced expressed sequence tags [ESTs] track). Nuclear RNAi machinery needs to overcome the intronic barriers for efficient and complete silencing of target sequences. Aquarius/EMB-4 can help overcome the intronic barriers to silencing and lead to spreading of 22G-RNAs along transcripts. Indeed, we observed that gene desilencing in both *hrde-1* and *emb-4* mutants correlated with 22G-RNA depletion (Figures 7E and 7F, yellow quadrant). Furthermore, the negative correlation between 22G-RNA abundance and mRNA levels is stronger when all exons of a gene are targeted by 22G-RNAs (Figures 7E and 7F, red line [all exons targeted] versus gray line [single exon targeted]) when considering *hrde-1* and *emb-4* mutant animals.

Taken together, our results support a model in which the RNA helicase Aquarius/EMB-4 is required to provide the co-transcriptional silencing complex access to nascent transcripts undergoing splicing (Figure 7G). We conclude that pre-mRNA processing is a natural and powerful barrier to co-transcriptional gene silencing.

## Discussion

Here we have identified the spliceosomal RNA helicase Aquarius/EMB-4 as an interactor of the germline nuclear Argonaute HRDE-1, which is required for heritable co-transcriptional gene silencing. We show that in *C. elegans*, Aquarius/EMB-4 expression is enriched in germ cell nuclei in adult-stage animals, which coincides with peak HRDE-1 expression in the germline. Our genetic experiments demonstrate that Aquarius/EMB-4 is required during the initiation step of silencing by HRDE-1 and that mutations in the helicase domain of Aquarius/EMB-4 are sufficient to impair its function in transcriptional gene silencing. Loss of Aquarius/EMB-4 leads to transcriptional desilencing of otherwise silenced genes, transposable elements, and piRNA sensor transgenes. We demonstrate that Aquarius/EMB-4 is specifically required to silence nascent transcripts undergoing splicing. In conclusion, we define a key interaction between sRNA pathways and the general transcription machinery. Aquarius/EMB-4 acts as a gatekeeper that permits sRNA pathways to monitor the nascent transcriptome. Interestingly, in a related article Claycomb's group reports that Aquarius/EMB-4 can also act together with the CSR-1 Argonaute protein in *C. elegans* (Tyc et al., 2017).

Previously, other factors involved in the nuclear sRNA-mediated transcriptional gene silencing have been identified in *C. elegans* and *D. melanogaster* through genetic screens (Ashe et al., 2012, Czech et al., 2013, Handler et al., 2013, Lee et al., 2012). These factors include the NRDE factors in *C. elegans* that are required for nuclear RNAi by an as yet unknown mechanism (Guang et al., 2008, Guang et al., 2010), as well as several histone methyltransferases and chromatin factors common to both nematodes and flies (Ashe et al., 2012, Shirayama et al., 2012). Much less is known of the events preceding chromatin level changes. In *C. elegans*, HRDE-1 and other nuclear RNAi factors not only are required for transcriptional gene silencing but also affect the biogenesis and/or the stability of 22G-RNAs, which are the effector sRNAs for nuclear gene silencing (Sapetschnig et al., 2015). One possible explanation is that in the absence of these factors, interactions between the nuclear Argonaute HRDE-1 and its target RNAs are abolished, leading to 22G-RNA destabilization. It is known that for the microRNA pathway, such Argonaute-target interactions are required to stabilize the associated microRNAs (Chatterjee and Grosshans, 2009, Chatterjee et al., 2011). Considering that numerous factors are required for effective HRDE-1 function, it is possible that RNA itself harbors intrinsic features refractory to HRDE-1 targeting.

### Intronic Barriers to Transcriptional Gene Silencing

Mammalian Aquarius binds to introns at a certain distance from the intron branch point, in a sequence-independent manner (Hirose et al., 2006). Even though the details of such interactions have not been studied in *C. elegans*, EMB-4 has been shown to bind nascent transcripts (Shiimori et al., 2012). Thus, it is reasonable to hypothesize that introns can influence the function of Aquarius/EMB-4 during transcriptional gene silencing. Indeed, when we reduced the number of introns from three to one in our piRNA sensor transgene, the absence of Aquarius/EMB-4 no longer resulted in desilencing of the transgene, showing that Aquarius/EMB-4 needs intronic sequences to function. Together with our observation that the single intron piRNA sensor accumulates several-fold more 22G-RNAs compared with a three-intron piRNA sensor, we propose that introns and/or factors interacting with introns are the inhibitory signals for HRDE-1 silencing.

### Transcription, Introns, and Silencing

Our results add to the growing body of evidence implicating co-transcriptional processes in sRNA-mediated gene silencing. In *S. pombe* an sRNA-mediated coTGS mechanism is required for the silencing of centromeric repeats (Allshire and Ekwall, 2015, Martienssen and Moazed, 2015). Several factors that interact with nascent transcripts and non-essential splicing factors are required for efficient coTGS (Bayne et al., 2008, Bayne et al., 2014). In addition, factors influencing the efficiency of RNA polymerase elongation are also important in coTGS mechanism in *S. pombe* (Kowalik et al., 2015). In plants, similar to our results, intron-containing transgenes are protected from nuclear RNAi pathway in comparison with the strongly silenced intronless transgenes (Christie et al., 2011). One hypothesis is that introns or factors interacting with introns are capable of blocking the nuclear RdRPs found in yeast and plants (Dumesic and Madhani, 2013, Vermeersch et al., 2010). In contrast, in the pathogenic yeast *Cryptococcus neoformans* unspliced introns are a signal for sRNA biogenesis and silencing of genes and transposable elements (Dumesic et al., 2013). Clearly, coTGS mechanisms in different organisms have evolved, one way or the other, to incorporate co-transcriptional processes for the regulation of gene silencing. Unlike in yeast and plants, animals do not possess nuclear RdRPs, and although *C. elegans* relies on RdRPs for sRNA amplification, this process occurs in the cytoplasm (Phillips et al., 2012). Nuclear processes that lead to coTGS in nematodes, flies, and mammals instead rely on the transport of sRNAs from cytoplasm to the nucleus by Argonaute proteins. Our results show that in animal coTGS pathways introns can pose a barrier to transcriptional gene silencing, and we provide evidence that the conserved spliceosomal helicase Aquarius/EMB-4 is required to remove these inhibitory signals.

## STAR★Methods

### Key Resources Table

**Table:** 

REAGENT or RESOURCE	SOURCE	IDENTIFIER
**Antibodies**		

Mouse monoclonal anti-EMB-4	This paper	5M19
Rabbit polyclonal anti-HRDE-1	(Ashe et al., 2012)	N/A
Rat monoclonal anti-OLLAS epitope tag	Novusbio	Cat#:NBP1-06713; RRID: AB_1625979
Rat monoclonal anti-alpha tubulin	Accurate Chemical	Cat#:YSRTMCA77P
Mouse monoclonal anti-alpha tubulin	SIGMA	DM1A; cat#: 9026; RRID: AB_477593
Rabbit polyclonal anti-Histone H3K9me3	Abcam	Cat#: ab8898; RRID: AB_306848
Goat anti-rabbit AlexaFluor 568	Molecular Probes	A11011; RRID: AB_143157
Goat anti-mouse AlexaFluor 647	ThermoFisher	A-21235; RRID: AB_141693

**Bacterial and Virus Strains**		

E. coli HB101	Caenorhabditis Genetics Center	N/A

**Critical Commercial Assays**		

TruSeq small RNA library preparation kit	Illumina	RS-200
NEBNext Ultra RNA library prep kit	NEB	E7530
Ribo-Zero rRNA Removal Kit	Illumina	MRZH11124

**Deposited Data**		

SILAC proteomics data of HRDE-1 IPs	This paper	Proteinexchange PXD004416
Small RNA and total RNA sequencing data	This paper	E-MTAB-4877
H3K9me3 ChIP-Seq data	This paper	E-MTAB-5662
HRDE-1 bound 22G-RNA data	(Sapetschnig et al., 2015)	GSE66344

**Experimental Models: Organisms/Strains**		

*C. elegans*: Strain SX1316 *mjIs144 II*	(Bagijn et al., 2012)	N/A
*C. elegans*: Strain SX2000 *mjIs144 II*; *hrde-1*(*tm1200*) *III*	(Ashe et al., 2012)	N/A
*C. elegans*: Strain SX2929 mjIs144 II; emb-4(qm31) V	This paper	N/A
*C. elegans*: Strain SX2930 mjIs144 II; emb-4(hc60) V	This paper	N/A
*C. elegans*: Strain SX3041 mjIs144 II; emb-4(sa44) V	This paper	N/A
*C. elegans*: Strain SX3073 mjIs588 II	This paper	N/A
*C. elegans*: Strain SX3074 mjIs144 II; hrde-1(tm1200) III; emb-4(sa44) V	This paper	N/A
*C. elegans*: Strain SX3078 mjIs588 II; hrde-1(tm1200) III	This paper	N/A
*C. elegans*: Strain SX3079 mjIs588 II; emb-4(qm31) V	This paper	N/A
*C. elegans*: Strain SX3117 emb-4(mjSi92)	This paper	N/A
*C. elegans*: Strain PD1504 ccSi1504 V	This paper	N/A
*C. elegans*: Strain SX3118 hrde-1(tm1200) III ccSi1504 V	This paper	N/A
*C. elegans*: Strain SX3179 emb-4(qm31) V ccSi1504 V	This paper	N/A
*C. elegans*: Strain VM285 neSi21	(Shirayama et al., 2012)	N/A

**Oligonucleotides**		

GFP mjIs144 RT-PCR Fwd: 5′-TCTGTCAGTGGAGAGGGTGA-3′	(Weick et al., 2014)	N/A
GFP mjIs144 RT-PCR Rev: 5′-TTTAAACTTACCCATGGAACAGG-3′	(Weick et al., 2014)	N/A
GFP mjIs588 RT-PCR Fwd: 5′-CGTACCATCTTCTTCAAG-3′	This paper	N/A
GFP mjIs588 RT-PCR Rev: 5′-GATGTTTCCGTCCTCCTT-3′	This paper	N/A
*cgh-1* RT-PCR Fwd: 5′-CCACCCCAGGAAGAATTCTC-3′	(Weick et al., 2014)	N/A
*cgh-1* RT-PCR Rev: 5′-GGTAAGTCTCGGCGTTTCTT-3′	(Weick et al., 2014)	N/A
*emb-4* RT-PCR Fwd: 5′-TTCGTCCCCTGTTCCATATC-3′	This paper	N/A
*emb-4* RT-PCR Rev: 5′-ATCGGCTTCTGGCCTAAAAT-3′	This paper	N/A
*act-3* RT-PCR Fwd: 5′-CCAAGAGAGGTATCCTTACCCTCAA-3′	This paper	N/A
*act-3* RT-PCR Rev: 5′-AAGCTCATTGTAGAAGGTGTGATGC-3′	This paper	N/A

**Recombinant DNA**		

pCFJ1416 (Psmu1:GFP2:smu1UTR) GFP2-GFP with altered codons and smu1 introns	This paper	(gift of Christian Frøkjær-Jensen)
CRISPR gRNA for emb-4 N-term 5′-CAAGAAGCCGTGGTGACTCG-3′	This paper	Dharmacon

**Software and Algorithms**		

MaxQuant quantitative proteomics software package	(Cox and Mann, 2008)	http://www.maxquant.org, version 1.3.0.5
RepeatMasker	(Smit et al., 2015)	http://www.repeatmasker.org/, version open-4.0.5
Cutadapt	(Martin, 2011)	Version 1.9.0
STAR aligner	(Dobin et al., 2013)	Version v2.5.1b
SAMtools	(Li et al., 2009)	v1.3
featureCounts	(Liao et al., 2014)	v1.5.0-p1
DESeq2	(Love et al., 2014)	v3.2.2
Segemehl	(Hoffmann et al., 2009)	version 2.0
SeqPlots	(Stempor and Ahringer, 2016)	http://przemol.github.io/seqplots/

### Contact for Reagent and Resource Sharing

Further information and requests for resources and reagents should be directed to and will be fulfilled by the Lead Contact, Eric A. Miska (eam29@cam.ac.uk).

### Experimental Model and Subject Details

*C. elegans* were grown under standard conditions at 20°C unless otherwise indicated. The wild-type strain was var. Bristol N2 (Brenner, 1974). E.coli strain HB101 was used as the food source on NGM plates (Stiernagle, 2006). Adult *C. elegans* animals were bleached to obtain synchronized L1 larvae population to grow synchronized animals used in experiments. Synchronized young adult animals were used in most experiments unless otherwise stated. All strains used are listed in Key Resources Table. Developmental stage of animals used in experiments are indicated in method details.

### Method Details

#### SILAC Proteomics

Bacterial and nematode growth conditions for SILAC experiments are previously described (Larance et al., 2011). Heavy (R10K8) labelled wild-type animals and medium (R6K4) labelled *hrde-1* mutant animals were grown to young adult stage, washed 3× with M9 buffer and lysed in native lysis buffer (10 mM Tris-HCl pH 7.5, 150 mM NaCl, 0.5 mM EDTA, 0.5% NP40, Roche complete protease inhibitor cocktail) by beat beating using zirconia beads and the PreCelys instrument (6,500 rpm, 3×30 s with 30 s intervals at 4°C). The lysate was kept on ice for 30 min and centrifuged for 10 min at 16,000 rcf at 4°C to remove insoluble material. BCA assay (Thermo Scientific) was used to determine protein concentration of the supernatant. 3-8 mg of total protein has been used for immunoprecipitations (IP) with 3 mg of anti-HRDE-1 antibody coupled dynabeads M270 (20 μg antibody / mg beads) for 1 hr at 4°C. Beads were washed 3× with wash buffer (10 mM Tris-HCl pH 7.5, 300 mM NaCl, 0.5 mM EDTA, Roche complete protease inhibitor cocktail) and equal amounts of beads from heavy and medium labelled IPs were mixed together prior to elution at the final wash. Elution was done by heating beads to 70°C for 10 min in LDS loading buffer. The eluted IP was loaded across multiple adjacent lanes (25 μl per lane) on 1 mm, 10-well, 4–12% (w/v) Bis-Tris NuPage gels using MES running buffer according to manufacturer's instructions but with the addition of 25 mM triscarboxyethylphosphine, and 50 mM N-ethylmaleimide in the LDS sample buffer. After electrophoresis at 150 V for 45 min, SYPRO Ruby staining was performed as per manufacturer's instructions (Invitrogen).

Protein bands of interest were excised and destained in 1 ml of 50% acetonitrile and 250 mM ammonium bicarbonate at room temperature for 45 min with shaking. The gel slice was dehydrated by incubation in 1 ml of 100% acetonitrile for 10 min at room temperature. All solution was carefully removed prior to the addition of 50 μl MS-grade trypsin (Promega) (12.5 ng/μl) in 100 mM NH4HCO3 and incubation overnight at 37°C. Peptides were extracted by the addition of 0.1 ml of 5% formic acid and incubation at 37°C for 1 hr. Peptides were further extracted by the addition of 0.1 ml of 100% acetonitrile and incubation at 37°C for 1 hr. The gel slice was completely dehydrated by the addition of 0.5 ml of 100% acetonitrile and incubation at 37°C for 10 min. The entire supernatant was then vacuum-dried.

#### LC-MS/MS and Analysis of Spectra

Using a Thermo Fisher Scientific Ultimate 3000 RSLCnano UHPLC, 15 μl of peptides in 5% (vol/vol) formic acid (final volume ∼10 μl) were injected onto an Acclaim PepMap C18 nano-trap column. After washing with 2% (vol/vol) acetonitrile, 0.1% (vol/vol) formic acid, peptides were resolved on a 50 cm Χ 75 μm C18 EasySpray reverse phase analytical column with integrated emitter over a gradient from 2% acetonitrile to 35% acetonitrile over 220 min with a flow rate of 200 nl/min. The peptides were ionized by electrospray ionization at +2.0 kV. Tandem mass spectrometry analysis was carried out on a Q-Exactive mass spectrometer (Thermo Fisher Scientific) using HCD fragmentation. The data-dependent acquisition method used acquired MS/MS spectra on the top 30 most abundant ions at any one point during the gradient. The RAW data produced by the mass spectrometer were analysed using the MaxQuant quantitative proteomics software package (Cox and Mann, 2008) (http://www.maxquant.org, version 1.3.0.5). The MaxQuant output has also been uploaded to the ProteomeXchange Consortium under the same identifier given above. This version of MaxQuant includes an integrated search engine, Andromeda. Peptide and Protein level identification were both set to a false discovery rate of 1% using a target-decoy based strategy. The database supplied to the search engine for peptide identifications was the combined *C. elegans* and *E. coli* Swissprot and Trembl databases downloaded on the 12th July 2012. The mass tolerance was set to 7 ppm for precursor ions and MS/MS mass tolerance was set at 20 ppm. Enzyme was set to trypsin (cleavage C-terminal to lysine and arginine) with up to 2 missed cleavages. Deamidation of Asn and Gln, oxidation of Met, pyro-Glu (with peptide N-term Gln), phosphorylation of Ser/Thr/Tyr, and protein N-terminal acetylation were set as variable modifications. N-ethylmaleimide on Cys was searched as a fixed modification. The output from MaxQuant provided peptide level data as well as protein group level data. We used the protein groups as defined by the Maxquant package (Cox and Mann, 2008).

#### HRDE-1 SILAC IP Experimental Design, Statistical Rationale, and Data Analysis

Three biological replicates were performed for SILAC-IP analysis of HRDE-1 and this level of replication was chosen based upon the variance detected in previous experiments using SILAC-IP analysis (Larance et al., 2012). To avoid disregarding low affinity binders, we used a low stringency cutoff such that a protein needed to have a H/M SILAC ratio >1 in two out of three biological replicates in our data analysis with MaxQuant to eliminate non-specific binding proteins. This yields excellent removal of environmental contaminants (keratins, trypsin, antibody, etc.) that do not incorporate stable isotopes.

#### HRDE-1/EMB-4 Co-immunoprecipitations and Western Blot Analysis

For HRDE-1 immunoprecipitations (Figures 1E, S1E, and S1F), animals were harvested 24 hours post-L4 stage in 30 mM HEPES, 100 mM potassium acetate, 2 mM magnesium acetate and 10% glycerol (DROSO buffer). To lyse the animals, samples were snap-frozen in liquid nitrogen as droplets and grinded to powder. *C. elegans* powder was then re-suspended in DROSO buffer supplemented with 0.1% NP-40 and further lysed by sonication. Lysates were subsequently cleared by centrifugation. 2 mg of proteins were used per IP at 4 mg/ml. For RNase treatment, lysates were either incubated with RNaseA or buffer for 30min at room temperature prior to addition of antibodies. 10 μg antibody (normal IgG: SantaCruz Biotech, sc-2027; anti-HRDE-1: Genomic Antibody Tech, custom (Kamminga et al., 2012)) was added to the lysates and incubated overnight at 4°C with rotation. 30 μl of proteinA/G-agarose beads (SantaCruz Biotech, sc-2003) were incubated for 2 hrs the next morning. Immunoprecipitates were washed 4 times with DROSO buffer and boiled with 2× sample buffer for 5 min to elute. Samples were then analysed by western blotting (Anti-Ollas: Novusbio, NMP1-06713).

For EMB-4 immunoprecipitations (Figure S1H), 750 μl of synchronized gravid adults were dounced using a metal wheaton dounce in DROSO ‘complete’ buffer (30 mM Hepes, 100 mM potassium Acetate, 2 mM Magnesium Acetate, 0.1% NP-40/Igepal, 2 mM DTT, 1 tablet/5mls Protease inhibitor (Roche), 1:100 Sigma Phosphatase Inhibitor 2, 1:100 Sigma Phosphatase Inhibitor 3.) until the worms and the embryos were no longer visible. Lysate was cleared by centrifugation for 10min at 13,000xg in a pre-cooled centrifuge (4°C). The concentration of the supernatant (total worm protein) was determined by Lowry assay using Bio-rad Lowry assay kit. Each IP was performed from 5 mg protein. Lysate was pre-cleared with 25 μl protein A/G agarose bead slurry (Santa Cruz Biotech, beads are equilibrated in DROSO complete buffer prior to use) for one hour at 4°C on a rotator. 5 μg (anti-Flag, Sigma Aldrich) or 50 μl of EMB-4 antibody (specificity of EMB-4 antibodies are shown in Figure S1E) or buffer alone (no antibody control) was added to each IP sample and incubated for two hours on a rotator at 4°C. Immune complexes were recovered using 50μl of a 50% slurry of Protein-A/G agarose beads (Santa Cruz Biotechnology) and washed 6x5min at 4°C with DROSO buffer. Protein was eluted from beads and denatured by incubation in Thermofisher 2x LDS sample buffer for 10min at 70°C. Input samples were prepared from the same lysate at a concentration of 2ug/ul using Thermofisher 2xLDS sample buffer and reducing agent. Proteins were resolved by SDS-PAGE on Criterion Precast gradient gels (4-15%, Biorad) and transferred to Hybond-C membrane(Amersham Biosciences). The membrane was incubated overnight at 4°C with either: (i) affinity purified anti-EMB-4 (1:200), or anti-FLAG (Sigma Aldrich, 1:1000) in PBST-5% milk solution (137 mM NaCl, 10 mM Phosphate, 2.7 mM KCl, pH 7.4, and 5% [w/v] dried milk). The membrane was incubated 2 h at room temperature with anti-mouse HRP-conjugated secondary antibody (Jackson Immunoresearch) diluted 1:1,000 in PBST and then visualized by Luminata Forte Western HRP substrate. Full blots of all IPs are available in Figure S6.

#### qRT-PCR Analysis of GFP Expression

cDNA was synthesised from purified and DNase treated total RNA (1μg) using the SuperScript II enzyme and random primers as described in the manual. qRT-PCR reactions were performed using Applied Biosystem Power SYBR Green Master Mix using primer sequences to amplify GFP sequence (primer 1: 5′-TCTGTCAGTGGAGAGGGTGA-3′, primer 2: 5′-TTTAAACTTACCCATGGAACAGG-3′) and the endogenous germline control gene *cgh-1* for normalisation (primer 1: 5′-CCACCCCAGGAAGAATTCTC-3′, primer 2: 5′-GGTAAGTCTCGGCGTTTCTT-3′).

#### qRT-PCR Analysis of *emb-4* Expression

cDNA was generated from 1mg of *C. elegans* total RNA using random hexamers with Superscript III Reverse Transcriptase (Invitrogen). qRT-PCR was performed using Applied Biosystems SYBR Green PCR Master mix with primers for *emb-4* (primer 1: 5′-TTCGTCCCCTGTTCCATATC-3′, primer 2: 5′-ATCGGCTTCTGGCCTAAAAT-3′) and for *act-3* (primer 1: 5′-CCAAGAGAGGTATCCTTACCCTCAA-3′, primer 2: 5′-AAGCTCATTGTAGAAGGTGTGATGC-3′).

#### Western Blot Analysis of EMB-4 Expression

Proteins were resolved by SDS-PAGE on Criterion Precast gradient gels (4-15%, Biorad). and transferred to Hybond-C membrane (Amersham Biosciences). The membrane was incubated overnight at 4°C with either: (i) affinity purified anti-EMB-4, or anti alpha-tubulin (Accurate Chemical) antibodies diluted to 1:2000, in PBST-5% milk solution (137 mM NaCl, 10 mM Phosphate, 2.7 mM KCl, pH 7.4, and 5% [w/v] dried milk). The membrane was incubated 1 h at room temperature with HRP-conjugated secondary antibodies (Jackson Immunoresearch) diluted to 1:5,000 in PBST and then visualized by Western Lightning ECL Kit from Perkin Elmer. Images were collected on a LAS-3000 Intelligent Dark-Box (Fujifilm).

#### Immunostaining of *C. elegans* Gonads and Embryos

For Figures 2C and S2A, gonads were excised from gravid adult worms in 1x PBS on poly-L-lysine coated slides, frozen and cracked on dry ice for longer than 10 minutes, and fixed at –20°C for 5 min in each of the following (15 minutes total) respectively; 100% methanol, 50% methanol/50% acetone, and 100% acetone. All sample incubations were performed in a humid chamber. Samples were washed 2x 5min with 1xPBS, then 2x 5 mins with 1xPBS / 0.1% Tween-20. Samples are then blocked for one hour in 1xPBS/0.1% Tween-20 / 3%BSA (PBST+BSA) at room temperature, and then incubated with primary antibody (1:500) overnight at 4°C. Slides were washed 3x for 10 minutes with PBST, and then incubated for 1 hour in PBST+BSA. Secondary antibodies were from Jackson Immunoresearch and Molecular Probes. Incubations with anti-mouse secondary antibodies were performed for one hour in PBST+BSA at room temperature. Slides were washed 3x for ten minutes in PBST, 3x for 5 minutes in PBS and then incubated with DAPI (1:2500) for 10 minutes at room temperature. Finally, slides were washed in PBS 3x for 5 minutes then mounted in Vectashield (Vector Labs). All images were collected using Nikon Ti-S inverted microscope with NIS Element and AR software.

For Figure S2B, gonad stainings were performed as described in (Hong et al., 2016). Adult animal gonads were dissected in dissection buffer (25mM HEPES pH7.4, 2mM MgCl_2_, 2mM CaCl_2_, 48mM KCl, 0.12 M NaCl, 0.2% Tween-20, 4mM levamisole) on cover slips and fixed 5min by equal volume of fixation buffer (25mM HEPES pH7.4, 2mM MgCl_2_, 2mM CaCl_2_, 48mM KCl, 0.12 M NaCl, 0.2% Tween-20, 4% formaldehyde). Glass cover slip were placed on poly-L-lysine coated glass slides and snap frozen in liquid nitrogen. Cover slips were removed to freeze crack the samples and glass slides were incubated in cold 50% aceton / 50% methanol for 10 min. Glass slides were washed 3X with 1% Triton-X100 PBS buffer, blocked with Image-enhancer (Lifesciences) for 20 min and blocked with blocking solution (PBS, 0.1% Tween-20, 1% BSA) for 20min. Slides were incubated with primary antibodies in blocking solution overnight at 4°C (α-EMB-4 5M19 1:100, α-HRDE-1 1:500), washed 3X with wash buffer (PBS, 0.1% Tween-20) and incubated for 2 hrs with secondary antibodies (α-mouse Alexa 647 1:750 (Lifetech) and anti-rabbit Alexa 568 1:750 (Lifetech). Slides were 3X with wash buffer and mounted using VectaShield DAPI mounting medium. Images were taken using a Leica SP8 confocal microscope using same laser settings between all slides imaged.

#### RNA Sequencing

Synchronised animals were grown to young adult stage at 20°C on HB101 seeded NGM plates. Animals were harvested and washed 3X in M9 buffer. Settled animals were mixed with Trisure reagent, bead beaten as described in proteomics experiments above and total RNA was isolated by a chloroform extraction.

For total RNA sequencing, Illumina Ribozero kit was used to remove ribosomal RNA from 1 μg of total RNA prior to library preparation. RNA sequencing libraries were prepared using NEB Next Ultra library preparation kit. Small RNA sequencing performed by treating 5 μg of total RNA with Epicentre 5′ polyphosphatase to remove the 5′ triphosphate from 22G-RNAs. After treatment, RNA is purified by phenol/chloroform extraction and 1 μg of RNA is used to prepare small RNA libraries using Illumina TruSeq small RNA library preparation kit. Ribosomal depleted RNA and small RNA libraries are sequenced using Illumina HiSeq 1500 platform.

#### RNA Sequence Analysis

The ce10/WS220 genome fasta file was obtained from the WormBase ftp server. Sequences for the piRNA sensor transgene and the piRNA sensor transgene with one intron were added as separate chromosomes when required. A GTF file containing annotations for genes and pseudogene for version ce10/WS220 of the *C. elegans* genome were downloaded from the UCSC table browser. To prevent multiple counts per read in the case of overlapping features, only the longest isoform of each gene was included in the analysis. A GTF file containing annotations for transposable elements was generated by running RepeatMasker (Smit et al., 2015) version open-4.0.5 in sensitive mode, run with rmblastn version 2.2.27+ using RepeatMasker database version 20140131, against the ce10/WS220 genome fasta file. “Simple_repeat” and “Low_complexity” annotations were excluded from the analysis. Raw fastq small RNA sequencing files were processed by removing the Illumina TruSeq adaptor sequence using cutadapt v1.9 (Martin, 2011), with parameters “--minimum-length 18 --discard-untrimmed -a TGGAATTCTCGGGTGCCAAGG”. Raw fastq RNA sequencing files were processed by removing the NEBNext adaptor sequence using cutadapt v1.9, with parameters “-a AGATCGGAAGAGCACACGTCTGAACTCCAGTCAC”. Adaptor-trimmed small RNA sequencing reads were aligned to the ce10/WS220 *C. elegans* genome using STAR v2.5.1b (Dobin et al., 2013), with parameters “--outFilterMultimapNmax 50 --winAnchorMultimapNmax 50 --outFilterMismatchNmax 0 --limitBAMsortRAM 31000000000 --alignIntronMax 1 --alignEndsType EndToEnd --outSAMtype BAM SortedByCoordinate --runThreadN 6 --outBAMsortingThreadN 6 --readFilesCommand 'gunzip -c'”. Adaptor-trimmed RNA sequencing reads were aligned to the ce10/WS220 *C. elegans* genome using STAR v2.5.1b, with parameters “--outFilterMultimapNmax 5000 --winAnchorMultimapNmax 10000 --outFilterMismatchNmax 2 --alignEndsType EndToEnd --outSAMtype BAM Unsorted --runThreadN --readFilesCommand 'gunzip -c'”. Aligned RNA sequencing reads were sorted and indexed using samtools v1.3 (Li et al., 2009). Counts against the annotations in the GTF files were generated with featureCounts v1.5.0-p1 (Liao et al., 2014), with parameters “-T 6 -M --fraction”. Normalised counts, variance-stabilised counts, fold change values, and adjusted p-values were obtained using DESeq2 v3.2.2 (Love et al., 2014), called through a custom script.

We also used exon-intron split analysis (EISA) (Gaidatzis et al., 2015) to characterize the gene expression changes detected between *hrde-1* or *emb-4* null and wild-type strains. Both exonic and intronic read counts were quantified using FeatureCounts (Liao et al., 2014). When using EISA, we processed the counts and the annotation files by following the procedures described by (Gaidatzis et al., 2015).

We calculated the 22G-RNA density using previously published small RNA sequencing data obtained from HRDE-1 immunoprecipitations in wild-type and mutant animals normalised to library size (Sapetschnig et al., 2015). We used a cut-off of 22G-RNA reads in wild-type / *hrde-1* mutant control ≥ 4 for filtering out 22G-RNA reads that were unspecifically binding to anti-HRDE-1 antibody. We then used the following calculation 22G-RNA density=# of 22G-RNA reads in HRDE-1 IP of gene A / RPKM of the gene A.

We carried out exon level sRNA differential expression by filtering out genes that have zero mapped reads in all samples and normalising the samples by sample size using the Median Ratio Method (Anders and Huber, 2010) implemented in the R package DESeq2 to adjust for factors like the coverage and sampling depth. Next, we log transformed the exon read counts for each gene, performed a two-sample t-test on each exon independently and adjusted the p-values of testing results by false discovery rate using the Benjamini & Hochberg method (Benjamini and Hochberg, 1995).

#### Histone H3K9me3 Chipmentation

H3K9me3 Chipmentation for wild-type, *hrde-1*(*tm1200*) and *emb-4*(*qm31*) young adult animals was performed according to (Schmidl et al., 2015) with slight modifications. Briefly, 50,000 animals were frozen and crushed in liquid nitrogen prior to 1% formaldehyde (SIGMA) fixation in 1XPBS. After 10 minutes of fixation at room temperature, 125 mM Glycine was added to quench the formaldehyde. Excess formaldehyde was washed twice with 1XPBS and once with the lysis buffer (50 mM HEPES pH:7.5, 150 mM NaCl, 0.1% TX100, 1 mM EDTA, protease and phosphatase inhibitors). The pelleted extract was resuspended in the lysis buffer and sonicated with a Bioruptor (15 SEC On, 90 SEC OFF, 10 cycles) to get 200-700 bps genomic DNA fragments at 4°C. 3 g of anti-H3K9me3 (Abcam, Ab8898) and 30 l Dynabeads Protein A (ThermoFisher) beads were incubated with 1 ml extract (Lysis buffer + 1% sarkosyl) for 12 hours on a rotating wheel at 4°C. The immunoprecipitated chromatin was washed with 150 mM, 500 mM and 1 M NaCl containing lysis buffer followed by LiCl and 1XTE pH:8.0 buffers, consecutively. Magnetic beads were resuspended in 1X tagmentation buffer with 1 μl Tn5 transposase Tagment DNA Enzyme (Illumina Nextera DNA Prep Kit). Tagmentation was performed at 37°C for 10 minutes. Beads were then washed twice with 1xTE pH:8.0. Two rounds of elution buffer (150 mM NaCl, 10 mM Tris pH:80, 1% SDS, 1 mM EDTA) was used to elute the immunoprecipitated DNA at 65°C. Eluted DNA was RNase and Proteinase K treated for 1 hour at 37°C and 12 hours at 65°C, respectively. De-crosslinked DNA was purified with Invitrogen PCR Cleaning Kit and DNA concentration was determined with Qubit HS DNA. In addition to ChIP DNAs, input DNAs were prepared from 5 ng purified DNA after sonication via ChIP-tagmentation (Illumina Nextera DNA Prep Kit).

#### Amplification and Sequencing of Chipmentation Libraries

1 μl of each library was amplified in a 20 μl qPCR reaction containing 0.2 M primers, 1X SyberGreen qPCR mix in StepOnePlus to determine the optimum number of PCR cycles in library preparation. Final libraries were prepared according to Illumina Nextera DNA Prep Kit with N cycles of PCR, where N is equal to the *Ct* value obtained from the qPCR analysis. Sequencing was performed in HiSeq2500 with using single end 50 bp reads.

#### Bioinformatic Analysis of H3K9me3 Chipmentation Data

Sequencing reads were aligned to the WS220/ce10 assembly of the *C. elegans* genome using Segemehl version 2.0 (Hoffmann et al., 2009) with 80% sequence similarity. SAMtools was used to convert aligned reads to BAM format. After mapping, the genome coverage was calculated for all individual sample replicates. Replicates were subsequently merged after normalisation by the library size factor s = c/g (with c: number of covered bases, and g: the size of the genome). Images for histone H3K9me3 enrichment on the piRNA sensor were prepared with SeqPlots (Stempor and Ahringer, 2016).

#### Transgenic Animals

The *mjIs588* allele was generated by removing the introns two and three from the GFP sequence in the plasmid pEM975 that is used to generate the *mjIs144* allele. New plasmid is inserted on Chr II using the previously described MosSCI method (Frøkjær-Jensen et al., 2008) into the same location as in *mjIs144* allele. *mjSi92* allele is generated by CRISPR tagging of endogenous *emb-4* N-term with the OLLAS epitope sequence using CRISPR gRNA (5′-CAAGAAGCCGTGGTGACTCG-3′) and the repair template plasmid pEM2058 using Cas9 protein and RNA injections as described in Paix et al. (Paix et al., 2015).

#### Structural Alignment of AQR and EMB-4

EMB-4 structure was determined by PHYRE-2 online prediction tool. Images were generated in Pymol (The PyMOL Molecular Graphics System, Version 1.8 Schrödinger, LLC) and mutagenesis was performed in Chimera (Yang et al., 2012).

### Quantification and Statistical Analysis

Quantification and statistical analysis of RNA-Seq, sRNA-Seq and H3K9me3 data is explained in the sections on these methods. Normalised counts, variance-stabilised counts, fold change values, and adjusted p-values were obtained using DESeq2 v3.2.2, called through a custom script. DESeq uses the negative binomial distribution to perform differential expression analysis. For exon level sRNA analysis, we log transformed the exon read counts for each gene, performed a two-sample t-test on each exon independently and adjusted the p-values of testing results by false discovery rate using the Benjamini & Hochberg method. For the analysis of H3K9me3 ChIP data, the genome coverage of reads was calculated for all individual sample replicates. Replicates were subsequently merged after normalisation by the library size factor s = c/g (with c: number of covered bases, and g: the size of the genome).

Number of animals used in microscopy experiments are indicated in relevant figure legends. Error bars indicate standard deviation unless otherwise stated in figure legends.

### Data and Software Availability

SILAC proteomics, RNA-Seq, sRNA-Seq and H3K9me2 ChIP data is deposited to public databases as indicated in the Key Resources Table.

The accession number for the SILAC proteomics data is Proteinexchange: PXD004416, the accession number for the RNA-Seq data is ArrayExpress: E-MTAB-4877, the accession number for the ChiP-Seq data is ArrayExpress: E-MTAB-5662.

## Author Contributions

A.A. and E.A.M. conceived and designed the study and wrote the manuscript. A.A., K.M.S., A.N., M.L., R.M., C.J.W., and A.C.B. performed the experiments. A.A., T.d.D., G.E.P., M.L., A.C.B., X.Z., P.M., J.E., and K.L.M.R. analyzed the data. A.A., R.M., A.N., and J.M.C. generated reagents. M.H., J.M.C., P.M., A.I.L., and E.A.M. provided expertise and feedback.

## Supporting Citations

The following references appear in the Supplemental Information: Frøkjær-Jensen et al., 2016, Han et al., 2009.

## Supplemental Information

Document S1. Figures S1–S6 and Table S1

Table S2. Fold Change Values of Genes that Show Significant Expression Change in Both HRDE-1 and EMB-4, Related to Figure 4

Document S2. Article plus Supplemental Information
