# Supplementary material for: The Helicase Aquarius/EMB-4 Is Required to Overcome Intronic Barriers to Allow Nuclear RNAi Pathways to Heritably Silence Transcription
Source: Dev Cell. 2017 Aug 7;42(3):241–255.e6. doi: 10.1016/j.devcel.2017.07.002 (PMC5554785; doi:10.1016/j.devcel.2017.07.002)
Supplement: Document S1. Figures S1–S6 and Table S1 [file mmc1.pdf]

Developmental Cell, Volume *42*

Supplemental Information

The Helicase Aquarius/EMB-4 Is Required to Overcome Intronic Barriers to Allow Nuclear RNAi Pathways to Heritably Silence Transcription

Alper Akay, Tomas Di Domenico, Kin M. Suen, Amena Nabih, Guillermo E. Parada, Mark Larance, Ragini Medhi, Ahmet C. Berkyurek, Xinlian Zhang, Christopher J. Wedeles, Konrad L.M. Rudolph, Jan Engelhardt, Martin Hemberg, Ping Ma, Angus I. Lamond, Julie M. Claycomb, and Eric A. Miska

SUPPLEMENTAL FIGURE LEGENDS

Figure S1. Related to Figure 1

(A) STRING database co-occurrence analysis based on the proteins used in the network in Figure 1B. Each square and the associated colour indicates the presence or absence and degree of conservation.

(B) Treefam data showing percent species with Aquarius/EMB-4 in each clade.

(C) Treefam tree based on sequence conservation of Aquarius/EMB-4 proteins in different model organisms (bootstrap values are indicated on branches).

(D) Assembly of splicing factors, Aquarius/EMB-4 and exon junction complex proteins during different stages of RNA splicing. Aquarius assembles during the B complex formation on intronic RNA and stays for the rest of the splicing reactions. EJC proteins assemble during Bact complex formation.

(E) Co-immunoprecipitation of OLLAS::EMB-4 with anti-HRDE-1 antibodies (IgG IP as negative control).

(F) Co-immunoprecipitation of OLLAS::EMB-4 with anti-HRDE-1 antibodies with or without RNase treatment (upper panel low RNase concentration, bottom panel high RNase concentration, IgG IP as negative control)

(G) Validation of the anti-EMB-4 antibody used in this study. The monoclonal anti-EMB-4 antibody (5M19-8) detects a single band at the size corresponding to EMB-4 (170kD) in wild type animals and no band is detected in the null *emb-4(hc60)* mutants (alpha-tubulin is used as loading control).

(H) Co-immunoprecipitations with anti-EMB-4 and anti-FLAG antibodies in animals expressing 3XFLAG::HRDE-1 and western blotting with anti-EMB-4 (upper panel) and anti-FLAG (bottom panel) antibodies (mock IP as negative control). (full length gel images of all immunoprecipitations are in Figure S6)

Figure S2. Related to Figure 1

(A) Immunostaining of *C. elegans* germline with anti-EMB-4 antibodies. Each panel highlights a different part of the germline.

(B) Co-staining of *C. elegans* germ cells with anti-EMB-4 and anti-HRDE-1 antibodies in wild type, *hrde-1* and *emb-4* mutant animals (three examples are shown for each genotype).

Figure S3. Related to Figure 2

(A) *mjIs144* transgene consists of *mex-5* promoter, GFP sequence with 3 synthetic introns, histone H2B sequence for nuclear localisation, piRNA target site for 21UR-1, *tbb-2* UTR and is inserted in Chr II by MosSCI single copy insertion. *mjIs144* transgene is fully silenced in wild type animals and de-silences in *hrde-1* and *emb-4* mutants.

(B) *ccSi1504* transgene consists of *smu-1* promoter, SV40 nuclear localisation signal, GFP sequence where all piRNA target sequences are removed (Frøkjær-Jensen et al., 2016), 4 *smu-1* introns, EGL-13 nuclear localisation signal, piRNA target site for 21UR-1, *smu-1* 3′UTR and is inserted in Chr V by MosSCI single copy insertion. *ccSi1504* is fully silenced in wild type animals and de-silences in *hrde-1* and *emb-4* mutants (numbers show individual animals assayed).

(C) qRT-PCR quantification of GFP levels in indicated mutant backgrounds with *ccSi1504* transgene (error bars=standard deviation of 3 biological replicates).

Figure S4. Related to Figure 3

(A) Domain structure of EMB-4 based on the domain structure of Aquarius (De et al., 2015).

(B) Crystal structure of Aquarius (domains are highlighted with same colours as in Figure S4A).

(C) Structural alignment of Aquarius (white) and EMB-4 (red).

(D) Superimposition of Vasa/AMPPNP/ssRNA with the crystal structures of Aquarius, EMB-4 and yeast Upf1-RNA. G884 residue of EMB-4 (shown in red) is embedded in close proximity to the putative ssRNA binding pocket.

(E) Western-blot showing EMB-4 protein abundance in wild type and *emb-4(sa44)* adult stage animals (alpha-tubulin as loading control).

Figure S5. Related to Figure 4.

(A) 21U-RNA (piRNA) abundance in animals with indicated genotypes (error bars represent standard deviation)

(B) Enrichment of genes in 22G-RNA density bins used in Figure 5H-I for germline and somatic 22G-RNA targets (Gu et al., 2009), for WAGO class 22G-RNA targets (Gu et al., 2009), for piRNA target genes (top 500, (Bagijn et al., 2012), for ERGO-1 target genes (Conine et al., 2010; Han et al., 2009), for ALG-3/4 target genes (Conine et al., 2010; Han et al., 2009) and for CSR-1 target genes (Claycomb et al., 2009).

Figure S6. Related to Figure 1 and Figure S1.

Full size blots of the immunoprecipitation experiments.

Table S1. Related to Figure 1. Comparison of HRDE-1 interactors with PIWI interactors. Proteins identified in HRDE-1 IPs (1st column) and their log2 fold enrichment values (2nd column, SILAC values heavy wild type IP / *hrde-1* mutant IP) and the number of replicates each protein was detected out of three replicates (2nd column in parentheses) are compared to proteins detected in *D. melanogaster* PIWI IPs (3rd column) and the functional RNAi screen results in *D. melanogaster* (last column).

Figure S1

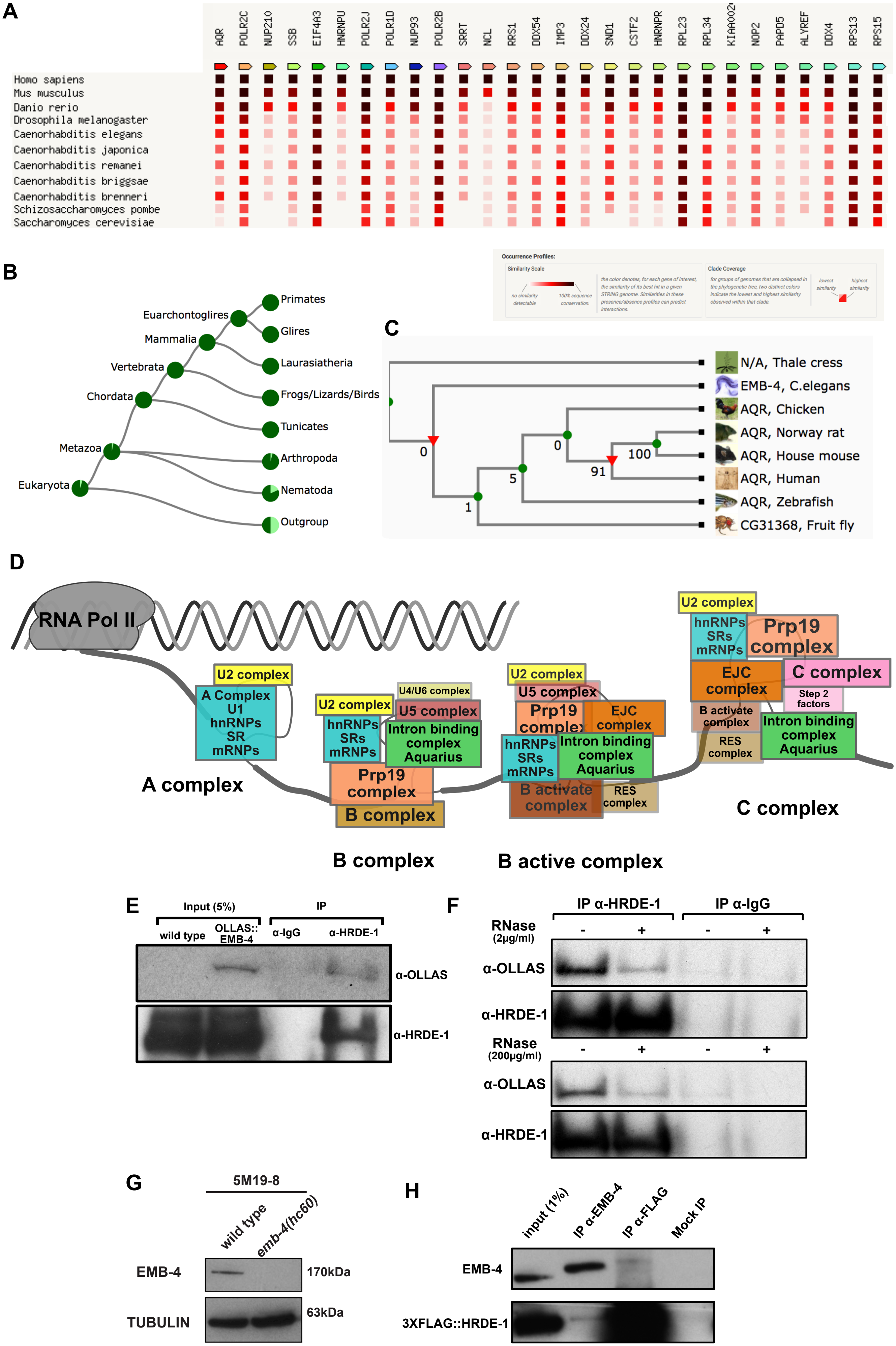

A
AQR
POLR2C
NUP210
SSB
EIF4A3
HNRNPU
POLR2J
POLR1D
NUP93
POLR2B
SRRT
NCL
RRS1
DDX54
IMP3
DDX24
SND1
CSTF2
HNRNPR
RPL23
RPL34
NOP2
PAPD5
ALYREF
DDX4
RPS13
RPS15
Homo sapiens
Mus musculus
Danio rerio
Drosophila melanogaster
Caenorhabditis elegans
Caenorhabditis japonica
Caenorhabditis remanei
Caenorhabditis briggsae
Caenorhabditis brenneri
Schizosaccharomyces pombe
Saccharomyces cerevisiae
Occurrence Profiles:
Similarity Scale
Clade Coverage
B
Primates
Euarchontoglires
Glires
Mammalia
Laurasiatheria
Vertebrata
Frogs/Lizards/Birds
Chordata
Tunicates
Metazoa
Arthropoda
Eukaryota
Nematoda
Outgroup
C
N/A, Thale cress
EMB-4, C.elegans
AQR, Chicken
AQR, Norway rat
AQR, House mouse
AQR, Human
AQR, Zebrafish
CG31368, Fruit fly
0
0
100
91
5
1
D
RNA Pol II
U2 complex
A Complex
U1
hnRNPs
SR
mRNPs
A complex
U4/U6 complex
U5 complex
SRs
Intron binding
complex
Aquarius
Prp19
B complex
EJC
B activate
RES
C complex
Step 2
factors
B active complex
E
Input (5%)
IP
wild type
OLLAS::
EMB-4
α-IgG
α-HRDE-1
α-OLLAS
F
IP α-HRDE-1
IP α-IgG
RNase
(2μg/ml)
(200μg/ml)
-
+
G
5M19-8
emb-4(hc60)
EMB-4
TUBULIN
170kDa
63kDa
H
input (1%)
IP α-EMB-4
IP α-FLAG
Mock IP
3XFLAG::HRDE-1

Figure S2

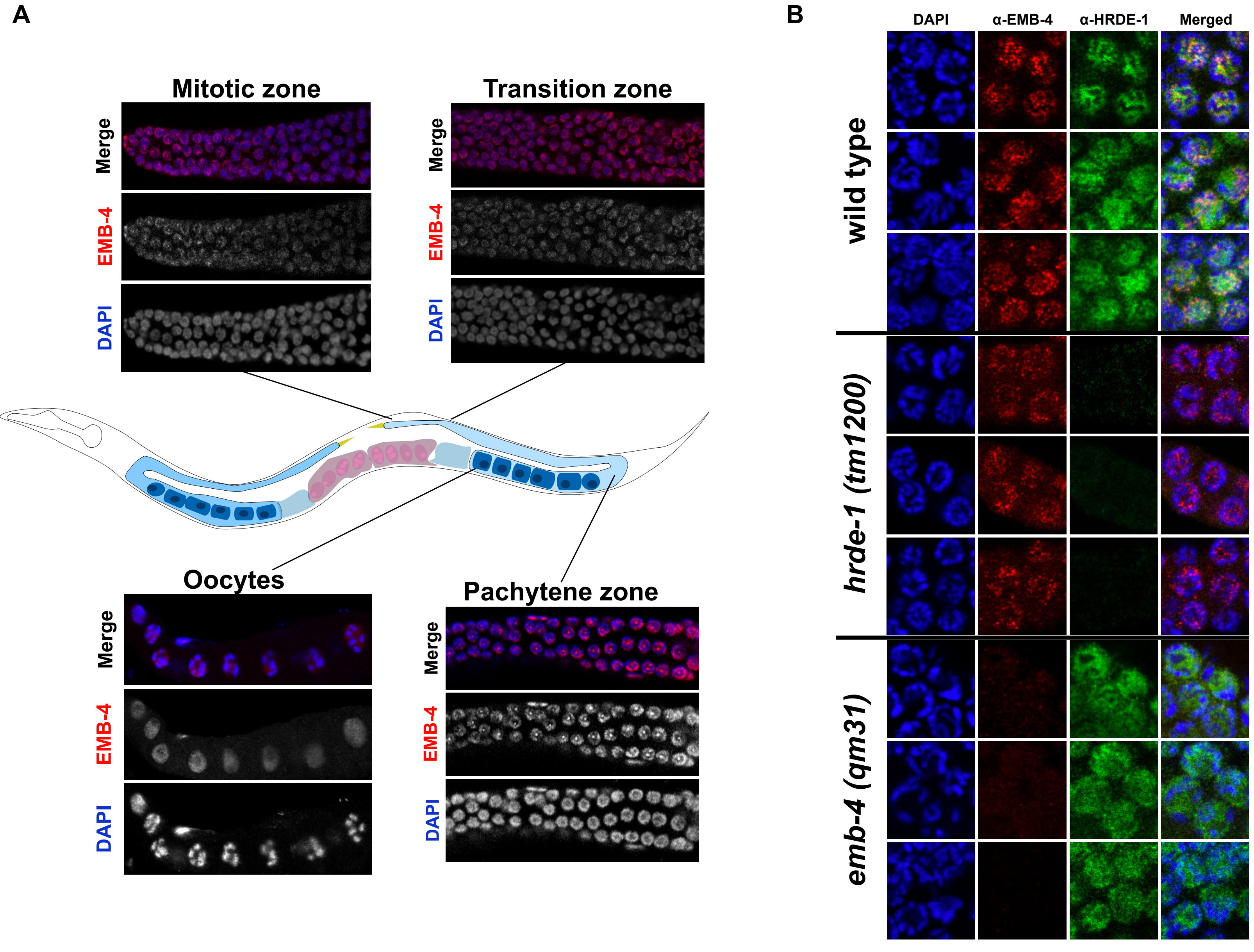
A
Mitotic zone
Transition zone
Merge
EMB-4
DAPI
Oocytes
Pachytene zone
B
DAPI
α-EMB-4
α-HRDE-1
Merged
wild type
hrde-1 (tm1200)
emb-4 (qm31)

Figure S3

A

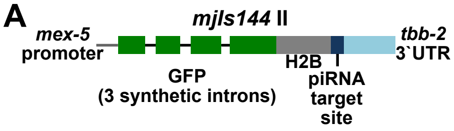

Wild type	*hrde-1 (tm1200)*	*emb-4 (qm31)*
OFF (20/20)	ON (20/20)	ON (20/20)

B

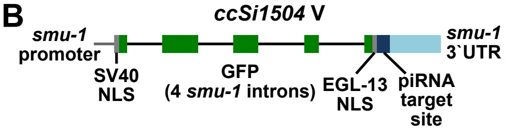

Wild type	*hrde-1 (tm1200)*	*emb-4 (qm31)*
OFF (20/20)	ON (20/20)	ON (20/20)

C

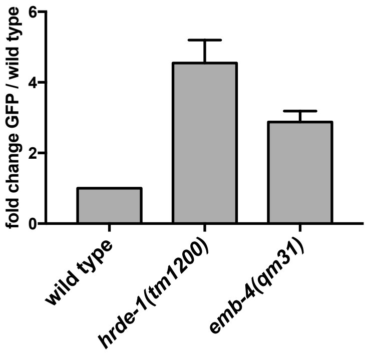

Figure S4

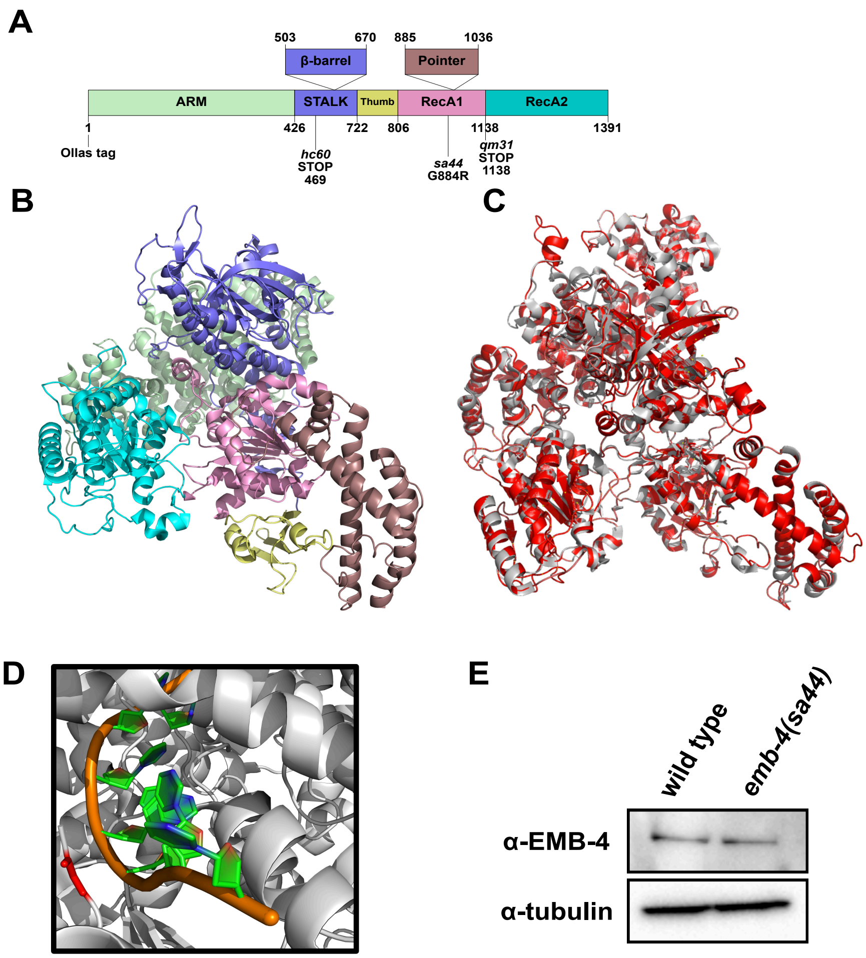
A
503
670
885
1036
β-barrel
Pointer
ARM
STALK
Thumb
RecA1
RecA2
1
426
722
806
1138
1391
Ollas tag
hc60
STOP
469
sa44
G884R
qm31
STOP
1138
B
C
D
E
wild type
emb-4(sa44)
α-EMB-4
α-tubulin

Figure S5

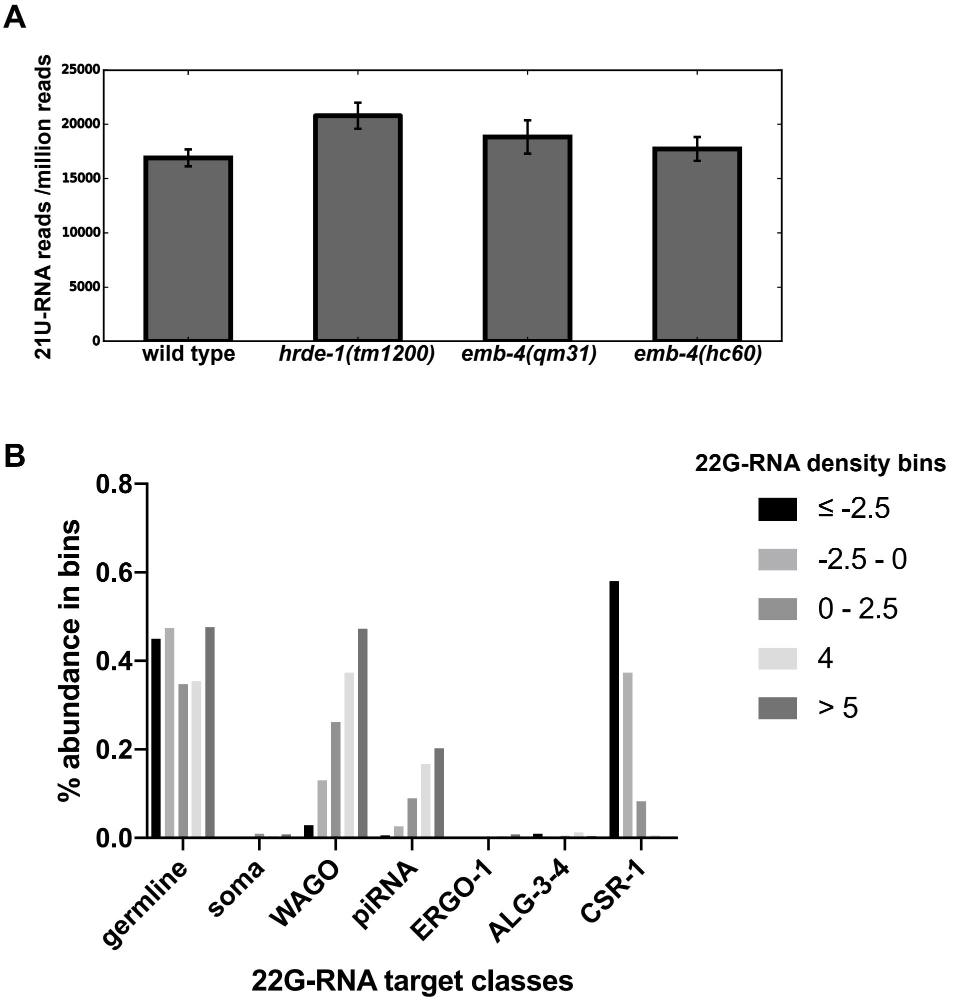
A
21U-RNA reads /million reads
25000
20000
15000
10000
5000
0
wild type
hrde-1(tm1200)
emb-4(qm31)
emb-4(hc60)
B
22G-RNA density bins
≤ -2.5
-2.5 - 0
0 - 2.5
4
> 5
% abundance in bins
0.8
0.6
0.4
0.2
0.0
germline
soma
WAGO
piRNA
ERGO-1
ALG-3-4
CSR-1
22G-RNA target classes

Figure S6

Figure 1E full blots

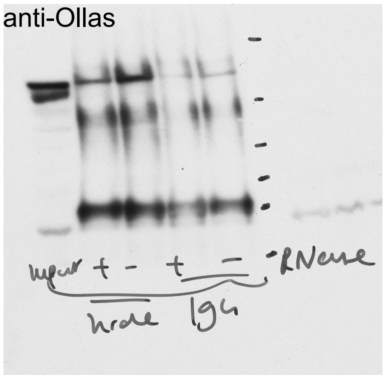

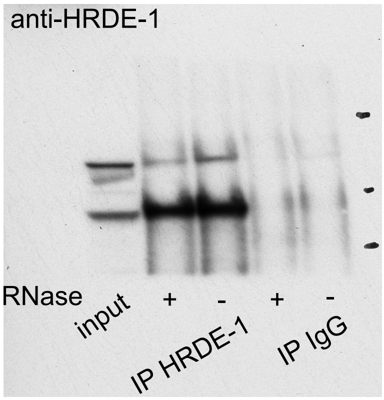

anti-OLLAS blot re-blotted with anti-HRDE-1

Figure S1E full blots

Figure S1F full blots

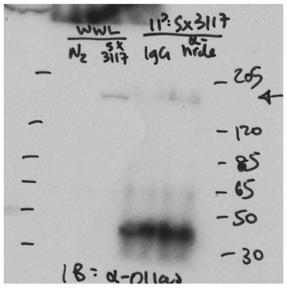

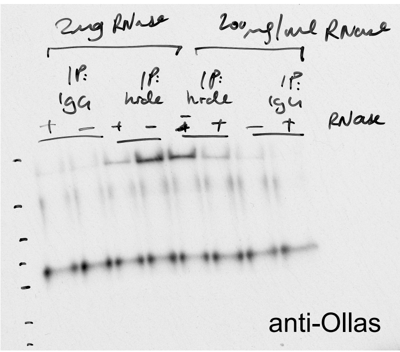

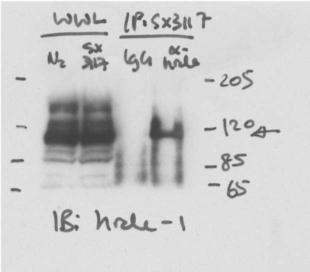

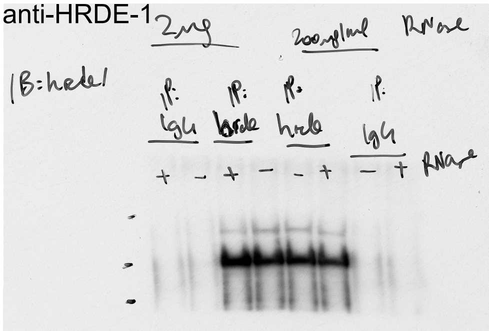

Figure S1H full blots

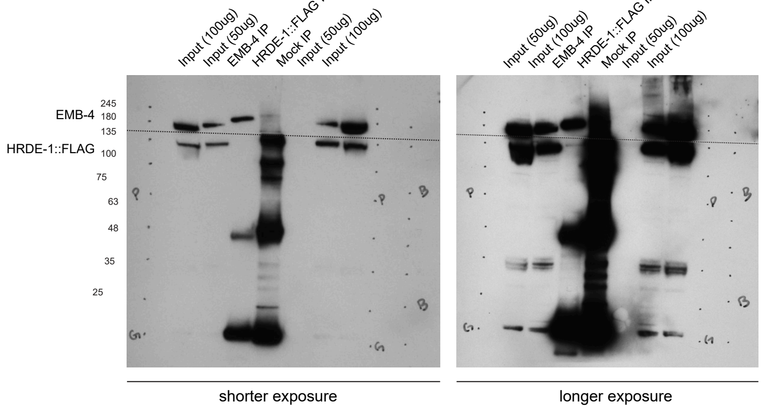

Table S1

Table S1. Comparison of HRDE-1 interactors with PIWI interactors			
	C. elegans HRDE-1 IP mean log2 fold enrichment (# of replicates detected)	*D. melanogaster* PIWI IP (Le Thomas et al., 2013)	*D. melanogaster* RNAi screen-germline TEs (Czech et al., 2013)
ALYREF (ALY-3, ALY)	0.68 (2/3)	3/3	4/4
RPL23 (RPL-23, RpL23)	0.43 (3/3)	3/3	1/4
DDX4 (GLH-2, VAS)	0.57 (2/3)	2/3	4/4
RPS15 (RPS-15, RpS15)	0.36 (3/3)	2/3	2/4
EIF4A3 (MEL-46, eIF4AIII)	0.74 (3/3)	2/3	2/4
AQR (EMB-4, CG31368)	0.73 (2/3)	2/3	1/4
NCL (K07H8.10, CG17108)	0.70 (3/3)	2/3	-
SND1 (TSN-1, Tudor-SN)	0.36 (3/3)	1/3	-
POLR2C (RPB-3, RpII33)	0.64 (2/3)	-	3/4
RPL34 (RPL-34, RpL34)	0.39 (2/3)	-	2/4
NOP2 (NOL-1, CG8545)	0.63 (2/3)	-	2/4
SRRT (E01A2.2, ARS2)	0.40 (2/3)	-	2/4
POLR2J (RPB-11, Rpb11)	0.54 (2/3)	-	2/4
POLR1D (F58A4.9, l(2)37Cg)	0.64 (2/3)	-	2/4
SSB (C44E4.4, La)	0.61 (2/3)	-	2/4
NUP210 (NPP-12, Gp210)	0.60 (2/3)	-	-
IMP3 (C48B6.2, CG4866)	0.27 (3/3)	-	-
RPS13 (RPS-13, RpS13)	0.42 (3/3)	-	-
RRS1 (RRBS-1, CG32409)	0.50 (2/3)	-	-
DDX24 (F55F8.2, CG9143)	0.54 (2/3)	-	-
KIAA0020 (PUF-12, PEN)	0.50 (2/3)	-	-
DDX54 (Y94H6A.5, CG32344)	0.55 (2/3)	-	-
PAPD5 (GLD-4, TRF4)	0.88 (2/3)	-	-
HNRNPU (Y71G10AL.1, CG30122)	0.29 (3/3)	-	-
HNRNPR (HRP-2, SYP)	0.59 (3/3)	-	-
NUP93 (NPP-13, Nup93)	0.46 (3/3)	-	-
CSTF2 (R09B3.2, CstF-64)	0.36 (2/3)	-	-
POLR2B (RPB-2, RpII140)	0.63 (2/3)	-	-
